# Supplementary material for: Transplantation of discarded livers following viability testing with normothermic machine perfusion
Source: Nat Commun. 2020 Jun 16;11:2939. doi: 10.1038/s41467-020-16251-3 (PMC7298000; doi:10.1038/s41467-020-16251-3)
Supplement: Supplementary file 1 — Supplementary Information [file 41467_2020_16251_MOESM1_ESM.pdf]

SUPPLEMENTARY INFORMATION

Mergental et al. Transplantation of discarded livers following viability testing with normothermic machine perfusion

Supplementary Table 1. Study livers overview

Liver number	Graft type	Inclusion criteria met								Viability criteria met	Total bile production (ml)	Liver transplanted	Additional Risk Scores		
		DRI	BAR	Steatosis⁺	CIT*	WIT§	Flush	Enzymes	TOTAL				UK-DLI	ET-DRI	UK-DCD
1	DCD	Y (2·7)	NA	N	Y 9:10	N (11)	N	N	2	Y	18	N (anatomy)~	2·29	3·31	4
2	DBD	N (1·6)	N (5)	N	N 7:20^	NA	N	N	0	Y	0	Y	1·04	2·38	-
3	DBD	Y (2·3)	Y (10)	N	N 5.59	NA	N	N	2	Y	46	Y	1·81	2·78	-
4	DBD	N (1·7)	Y (11)	N	Y 13:13	NA	N	N	2	Y	64	Y	1·05	2·12	-
5	DBD	Y (2·5)	N (3)	N	N 10:25	NA	N	N	1	Y	>99	Y	1·23	2·92	-
6	DBD	Y (2·1)	N (3)	N	Y 14:50	NA	N	N	2	Y	60	Y	1·18	2·42	-
7	DBD	Y (2·1)	N (3)	N	N 6:30	NA	N	Y (1812)	2	Y	>99	Y	0·99	2·52	-
8	DCD	Y (3·0)	N (3)	N	Y 9:33	N (23)	N	N	2	Y	90	Y	1·98	3·78	7
9	DCD	Y (2·1)	NA	N	N 6:00	N (20)	N	Y (1383)	2	N	>99	N (lactate drifted)	1·67	2·92	5
10	DCD	Y (2·9)	NA	N	N 7:16	N (20)	N	N	1	N	10	N	1·72	3·41	7
11	DBD	N (1·6)	N (4)	N	N 5:24	NA	N	Y (1041)	1	Y	60	Y	1·60	1·92	-
12	DCD	Y (2·9)	NA	N	Y 9:59	N (25)	N	N	2	N	0	N	2·00	3·68	5
13	DCD	Y (2·2)	N (2)	N	N 6:29	N (17)	Y	N	2	Y	0	Y	1·42	3·03	4
14	DCD	Y (2·9)	N (5)	N	N 7:09	Y (40)	N	N	2	Y	0	Y	1·70	3·41	10
15	DCD	Y (3·2)	N (1)	N	N 5:32	N (22)	N	N	1	Y	>99	Y	1·88	3·63	7
16	DBD	N (1·8)	N (8)	Y (50%)	N 11:55	NA	N	N	1	Y	0	Y	1·41	2·68	-
17	DBD	N (1·9)	N (6)	N	Y 12:00	NA	Y	N	2	Y	0	Y	1·10	2·22	-
18	DCD	Y (2·1)	N (7)	N	N 7:44	Y (35)	N	N	2	Y	75	Y	1·95	3·19	8
19	DCD	Y (2·5)	N (2)	N	N 7:00	Y (46)	N	N	2	Y	>99	Y	1·57	3·14	8
20	DCD	Y (3·1)	N (2)	N	N 5:54	N (19)	Y	N	2	Y	48	Y	2·65	3·72	4
21	DCD	Y (3·2)	N (4)	N	N 6:52	N (23)	N	N	1	Y	15	Y	2·52	4·51	9
22	DBD	Y (2·3)	NA	N	N 8:36	NA	N	Y (1143)	2	Y	18	N (donor cancer)	1·13	4·25	-
23	DCD	Y (3·1)	N (3)	N	Y 10:00	N (18)	Y	N	3	Y	>99	Y	2·33	3·50	6
24	DBD	Y (2·0)	NA	Y (80%)	Y 12:00	NA	N	N	3	N	0	N	1·18	2·96	-
25	DBD	Y (2·4)	NA	N	Y 13:24	NA	Y	N	3	N	2	N	1·38	2·77	-
26	DBD	N (1·2)	NA	N	N 6:00	NA	Y	Y (1811)	2	Y	30	N (lactate drifted)	0·72	2·15	-
27	DBD	N (1·9)	NA	N	N 11:55	NA	Y	N	1	N	3	N	0·93	2·71	-
28	DCD	Y (3·8)	N (4)	N	N 5:34	N (20)	N	N	1	Y	56	Y	2·77	3·76	9
29	DBD	N (1·7)	N (3)	Y (60%)	N 6:15	NA	N	N	1	Y	15	Y	1·16	2·03	-
30	DBD	Y (2·1)	N (3)	N	N 7:46	NA	N	N	1	Y	>99	Y	1·35	2·76	-
31	DBD	Y (2·1)	N (3)	N	N 7:33	NA	N	N	1	Y	63	Y	1·01	2·68	-

Abbreviations: BAR, balance of risk score; DRI, donor risk index; Y, Yes; N, No; NA, not applicable; CIT, cold ischaemic time; WIT, donor warm ischaemic time (DCD only); DCD, circulatory death donor; DBD, brainstem death donor; UK-DLI, United Kingdom donor liver index; ET-DRI, Eurotransplant Zone donor risk index; UK-DCD, United Kingdom DCD score

Notes: ⁺Steatosis assessment from the local pathologist at the centre that initially accepted the liver. *Time expressed in hours:minutes. § Donor warm ischaemic time is defined as the period from the systolic blood pressure decrease below 50mmHg to commencing the aortic cold flush and is expressed in minutes. Highlighted inclusion criteria cells designate the criterion was met (dark grey highlights the main criterion used for the study inclusion, light grey additional criteria met). ^The liver was a fast-track offer from another centre following the initial recipient intra-operative death, where the short notice precluded commencement of the transplantation within 12 hours of CIT. ~The liver was not used because of multiple arterial reconstructions and poor vessels quality. Donor risk index is calculated from age, race, cause of death, height and the predicted cold ischaemic time (Feng *et al* 2006); BAR is calculated using model for end-stage liver disease score (MELD), whether or not the recipient is having a re-transplant or is on intensive care, recipient age, donor age and cold ischaemic time (Dutkowski *et al* 2011); UK-DLI is calculated using donor age, sex, height, donor type, bilirubin, smoking history, and whether the liver was split (Collett *et al* 2017); ET-DRI is calculated using donor age, cause of death, whether whole or split liver, regional or national share, gamma glutamyl transferase and whether a rescue offer (Braat *et al* 2012); UK-DCD score is calculated using donor age, donor body mass index, duration of functional warm ischaemic time, cold ischaemic time, recipient age, MELD score, and re-transplant status (Schlegel *et al* 2018). Additional risk score data supplied by RW Laing and steatosis percentages supplied by DAH Neil.

Supplementary Table 2. Histology findings

Liver Number	Liver Type	Steatosis (Prior to NMP)		Glycogen depletion			Bile duct injury		Other
		LARGE DROPLET	SMALL/MED DROPLET	Pre-NMP	4hrs NMP	Post NMP	Pre-NMP	After liver implantation	
1	DCD	nil	mild	severe	severe	severe	moderate	not applicable	Moderate to severe siderosis
2	DBD	nil	nil	severe	severe	moderate	min	none	
3	DBD	nil	mild	mild	mild	moderate	mild	mild-mod	
4	DBD	nil	severe	mild	mild	mild	mild-moderate	mod-severe*	
5	DBD	nil	mild	nil	nil	nil	minimal	mod-severe#	
6	DBD	mild	moderate	nil	mild	nil	minimal	minimal	Minimal siderosis
7	DBD	mild	severe	severe	severe	nil	minimal	minimal	
8	DCD	mild	mild	mild	nil	nil	mild-moderate	mild -moderate*	
9	DCD	nil	severe	severe	severe	moderate	mild-moderate	not applicable	Mild siderosis
10	DCD	mild	severe	mild	mild	moderate	mod	not applicable	
11	DBD	mild	severe	severe	severe	mild	mild	moderate	
12	DCD	nil	moderate	severe	severe	severe	mod-severe	not applicable	
13	DCD	nil	nil	nil	nil	nil	minimal	mild	
14	DCD	nil	mild	moderate	mild	mild	mod-severe	severe#	
15	DCD	mild	moderate	severe	severe	mild	mild	mod-severe	Mild siderosis
16	DBD	mild	severe	severe	severe	moderate	mild-moderate	moderate	Mild siderosis
17	DBD	nil	nil	nil	nil	nil	severe	severe	Minimal siderosis
18	DCD	nil	mild	severe	moderate	severe	mod-severe	severe	
19	DCD	nil	nil	moderate	mild	nil	moderate	minimal	
20	DCD	nil	mild	severe	moderate	moderate	minimal	mild-mod#	Moderate siderosis
21	DCD	mild	moderate	mild	mild	mild	mild	severe	
22	DBD	nil	nil	mild	nil	nil	minimal	not applicable	Not transplanted because malignancy found in donor, perfusion stopped at 4 hours.
23	DCD	mild	moderate	mild	nil	moderate	mild	severe	
24	DBD	severe	severe	moderate	severe	severe	minimal	not applicable	
25	DBD	mild	severe	mild	nil	moderate	mild	not applicable	Steatohepatitis + mild fibrosis
26	DBD	nil	severe	moderate	severe	severe	mild	not applicable	
27	DBD	moderate	moderate	mild	severe	moderate	mild	not applicable	Steatohepatitis + mild/moderate fibrosis
28	DCD	nil	nil	moderate	nil	mild	moderate	severe#	
29	DBD	nil	severe	severe	severe	moderate	mild	mod-severe	Mild fibrosis, Mild siderosis
30	DBD	nil	mild	severe	severe	mild	moderate	mild-moderate	Minimal siderosis
31	DBD	mild	moderate	mild	mild	mild	nil	no biopsy taken	Minimal siderosis

Abbreviations: NMP, normothermic machine perfusion; DCD, donation after circulatory death; DBD, donation after brainstem death

Note: Large droplet fat is defined as macrovesicular steatosis with a single large fat droplet within the hepatocyte cytoplasm displacing the nucleus. Small and medium droplet macrovesicular steatosis is defined as fat droplets, usually multiple, within the cytoplasm of the hepatocyte which do not displace the nucleus. Both types of steatosis and glycogen depletion are graded by the % of hepatocytes containing fat droplets or not containing glycogen respectively based on thirds, with up to 5% being considered negative ie. none: 0-5%; mild: 5-33%; moderate: 34-67%; severe: >67%. Injury to extrahepatic bile ducts was determined by grading injury to deep peribiliary glands (0-3), stromal nuclear loss (0-3) and loss of nuclei in the media of arteries/arterioles (0-3), the extent of haemorrhage (0-4) and presence of thrombi (Y/N) (op den Dries et al 2014 and Hansen et al 2012). An overall bile duct injury grade was assigned based on the severity/presence of each eg. minimal = mild of one or two features, severe = moderate to severe in all etc. Rows with gray background designates transplanted livers. *Designates livers that developed anastomotic biliary strictures. #Designates livers that developed symptomatic non-anastomotic biliary strictures.

Supplementary Table 3. Post-transplant recovery and follow up

Transplant Number	Donor type	Total preservation time (hours:minutes)	Post-reperfusion syndrome	Early allograft dysfunction	Peak ALT /AST	Renal replacement therapy (days)	Clavien-Dindo complication grade	ITU stay	In hospital stay	Anastomotic / Non-anastomotic biliary strictures	Graft survival up to the last follow up (days)	Patient survival up to the last follow up (days)	Re-Tx
1	DBD	18:55	No	Yes	1176/3165	No	2	2	8	No/No	784	784	No
2	DBD	17:05	No	No	507/322	No	2	4	8	No/No	647	647	No
3	DBD	21:07	No	No	230/247	No	2	2	7	Yes/No	641	641	No
4	DBD	20:05	No	Yes	688/641	No	4	4	30	No/Yes*	225	708	Yes
5	DBD	23:50	No	No	614/1038	No	1	2	11	No/No	656	656	No
6	DBD	24:13	No	No	289/215	No	2	6	8	No/No	472	472	No
7	DCD	17:03	Yes	Yes	921/2510	No	3	3	7	Yes/No	634	634	No
8	DBD	17:09	No	No	824/1095	No	2	4	10	No/No	456	456	No
9	DCD	25:32	Yes	Yes	306/216	No	2	6	17	No/No	620	620	No
10	DCD	17:29	Yes	Yes	2339/3612	Yes (3)	4	3	19	No/Yes	375	650	Yes
11	DCD	18:17	Yes	No	529/716	No	2	2	10	No/No	611	611	No
12	DBD	21:48	No	Yes	477/1543	No	2	4	13	No/No	558	558	No
13	DBD	17:27	No	No	166/240	No	2	4	10	No/Yes^	517	517	No
14	DCD	14:38	Yes	No	594/331	No	2	3	11	No/Yes^	513	513	No
15	DCD	16:17	No	No	392/495	No	2	3	9	No/No	561	561	No
16	DCD	11:21	Yes	No	57/166	Yes (37)	4	38	47	No/Yes	120	509	Yes
17	DCD	13:59	Yes	No	394/677	No	2	3	10	No/Yes^	525	525	No
18	DCD	24:00	Yes	No	255/423	Yes (29)	4	18	32	No/Yes^	442	442	No
19	DCD	14:34	No	No	338/327	No	3	3	17	No/Yes	417	417	Yes
20	DBD	15:03	Yes	Yes	2074/2836	No	2	3	14	No/No	390	390	No
21	DBD	23:46	Yes	No	273/626	Yes (3)	4	7	10	No/No	403	403	No
22	DBD	18:53	No	No	827/1385	No	2	4	6	No/No	423	423	No

Abbreviations: ALT, alanine aminotransferase; AST, aspartate aminotransferase; DBD, donor following brainstem death; DCD, donor following circulatory death; ITU, intensive treatment unit; Re-Tx, re-transplant required.

Note: *Designates patient with hepatic artery thrombosis requiring early revascularisation. ^Designates asymptomatic non-anastomotic biliary strictures without cholestasis.

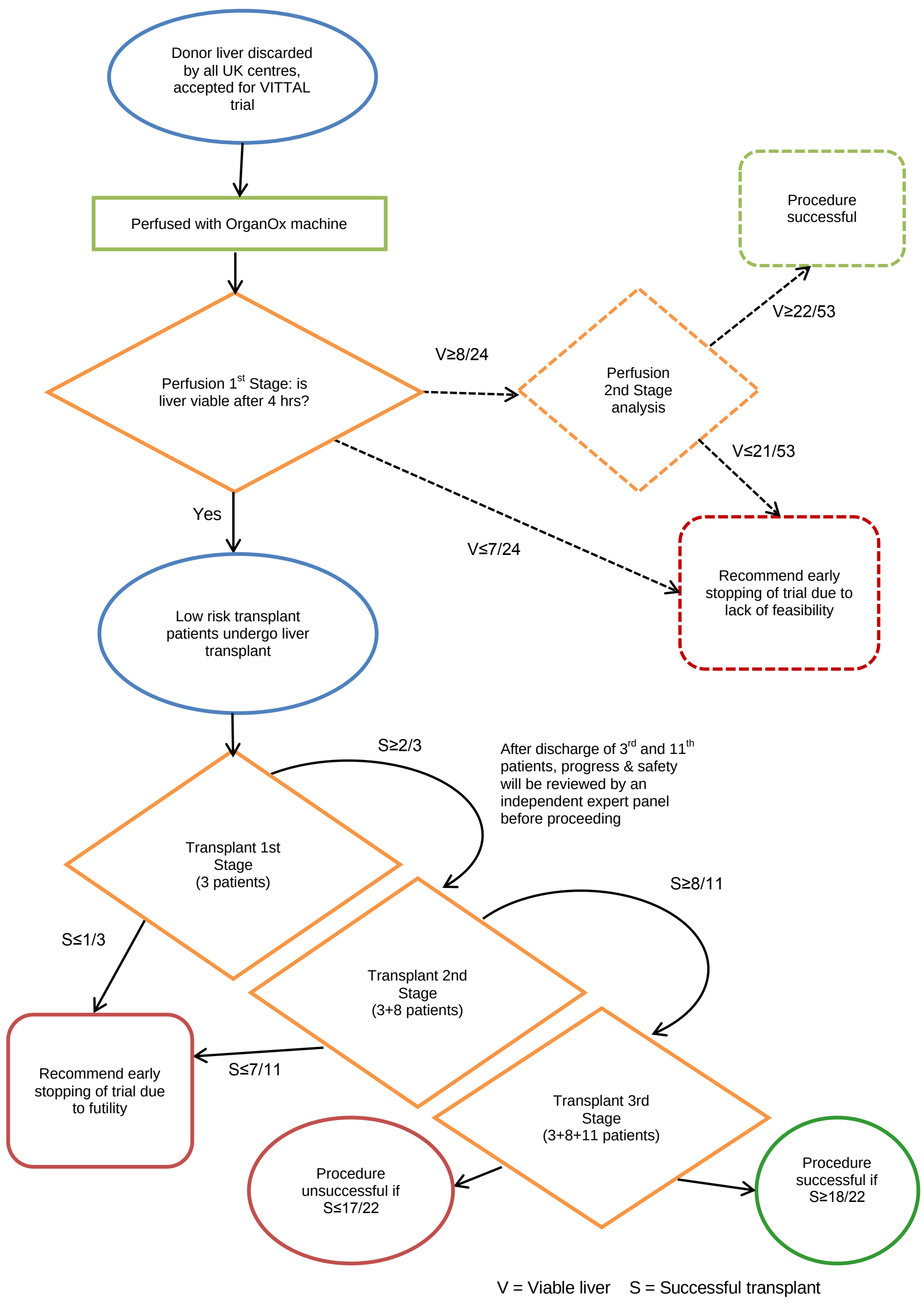

Supplementary Figure 1. The study schema

This study was a prospective, open label, phase 2 adaptive single-arm trial comprising high-risk livers meeting two-tier inclusion criteria. The trial was powered with an emphasis on the feasibility of the intervention using NMP and recipient safety. In terms of the intervention feasibility, the aim was to achieve an organ recovery rate of at least 50%, with a rate of 30% or less being considered unacceptable. Using a two-stage design, with an interim assessment after 24 livers (continuing if ≥8 livers were recovered), a sample size of up to 53 livers undergoing NMP might be required, with target alpha (one-sided) of 0.05 (actual alpha = 0.047) and target beta of 0.1 (actual beta = 0.098) . NMP was considered feasible for organ recovery if at least 22 livers were recovered from 53 perfused. Though the two statistical inferences are assessing different hypotheses (safety and feasibility), they are linked

as 22 transplants are required for the safety testing of the procedure, which is also the minimum number required out of 53 perfused livers to be considered feasible.

Using an optimal three-stage adaptive design with two interim assessments after 3 patients (requires ≥2 successes) and 11 patients (≥8 successes), a sample size of 22 patients was required, with alpha (type I error) and beta (type II error) of 0.2. As this was an early phase (non-definitive) trial to assess the safety of this procedure, a relaxed one-sided alpha was used to attain an achievable sample size within the trial duration and cost constraint. The approach was considered successful if there were at least 18 successes out of 22 transplants

VITTAL

Viability testing and transplantation of marginal livers

An open label, non-randomised, prospective, single arm trial, using normothermic machine liver perfusion NMLP to test viability and transplantation of marginal livers

STUDY PROTOCOL

**Protocol Version**	V2.0c 01 October 2018
**Sponsor Number (RG Number)**	RG 15-240
**EUDAMED Number**	CIV-GB-16-08-016567
**ClinicalTrials.gov Number**	NCT02740608
**CAS Number**	HE 1012
**IRAS Number**	206074
**Sponsor**	The University of Birmingham
**Chief Investigator**	Professor Darius Mirza

UNIVERSITY OF BIRMINGHAM

@CRCTU Cancer Research UK Clinical Trials Unit

NHS National Institute for Health Research

OrganOx living organs for life

wellcome trust

University Hospitals Birmingham NHS Foundation Trust

AMENDMENTS

The following amendments and/or administrative changes have been made to this protocol since the implementation of the first approved version

Amendment number	Date of amendment	Protocol version number	Type of amendment	Summary of amendment
1	02 December 2016	2.0	Substantial amendment	The criteria for liver transplantation have been amended (section 4.3 Lactate must be met along with 2 of the other criteria).<br>Order of co-objectives amended in the Synopsis to match those in Section 13.<br>Updated patient and liver pathway added (section 6)<br>Updated schedule of events<br>Updated sample collection details and checklist (section 6 and appendix 6)<br>Wording of co-objective B updated (section 8)
2	12 January 2017	2.0 a	Substantial amendment	Typographical error corrected- Criteria for Transplantation (pH, HA flow and PV flow) on page 11 updated to read 'greater than or equal to' to match Criteria for Transplantation on page vi.
3	12 July 2017	2.0 b	Non-substantial amendment	Amendment made to include an explicit trial schema, interim safety assessment review using patient hospital release data in order to reduce length of time trial is closed to recruitment, and criteria to be used to select cases for the contemporaneous control comparison group. Also to update the process for ordering recipient-matched blood
4	01 October 2018	2.0c	Non-substantial amendment	Amendment made to update the Confidentiality and Data Protection section according to General Data Protection Regulation changes

TRIAL SYNOPSIS

**Title**	Viability testing and transplantation of marginal livers.<br>An open label, non-randomised, prospective, single arm trial, using normothermic machine liver perfusion NMLP to test viability and transplantation of marginal livers	
**Trial Design**	The proposed design is an open label, non-randomised, prospective, single arm trial, performing normothermic machine liver perfusion (NMLP) on rejected livers until we reach 22 clinical transplants. It is designed with two linked components to assess;<br>• the feasibility of NMLP in rejected organ recovery using an established 2-stage design (Simon, 1989)<br>• successful transplantation using an optimal 3-stage design (Chen, 1997).	
**Trial duration**	It is anticipated that recruitment will take 15 months. All patients will be followed up for 6 months post-transplant. This recruitment is based on an average of 1-2 patients being recruited each month from the Queen Elizabeth Hospital, Birmingham, UK	
**Objectives and Outcome Measures**		
**Co-Primary**	**Objective**	**Outcome Measure**
	1a Establish the feasibility of NMLP as a means to increase the number of transplantable livers.	1a “Rescue-rate” i.e. the number of rejected livers that can be salvaged and used for clinical transplantation.
	1b Achievement of successful transplantation using rejected liver grafts	1b 90-day patient survival
**Secondary**	**Objective**	**Outcome Measure**
	Assess the liver graft function following transplantation (by incidence of primary non-function, and early allograft dysfunction	• Liver function tests<br>• 90-day graft survival<br>• 12-month patient and graft survival<br>The secondary endpoints and other outcome measures will be compared with a contemporary matched recipient group
	Assess the physiological response to reperfusion of the perfused grafts	Post-reperfusion syndrome (Defined as a decrease in mean arterial pressure (MAP) of more than 30% from the baseline value for more than one minute during the first five minutes after reperfusion (assessed in the context of inotrope use)).

	To compare clinical course of transplantation using rejected liver grafts with historical controls carried out with 'transplantable' liver grafts	• Adverse event rates and severity, graded according to the Clavien-Dindo classification<br>• Primary graft non-function rate<br>• 90-day graft survival<br>• 12-month patient and graft survival<br>• Incidence of non-anastomotic biliary strictures<br>• Requirement of renal replacement therapy
	Identify impact upon quality of life after transplantation with these liver grafts	• Quality of life analysis using EQ-5D-5L questionnaire
**Exploratory**	**Objective**	**Outcome Measure**
	Identify novel biomarkers that are indicative of liver quality and function	This will be performed through careful analysis of biological samples and through correlation of findings with perfusion outcome and if transplanted, clinical outcome
	Identify the feasibility of using NMLP to increase the number of liver transplants	Health economic analysis
**Main Inclusion and Exclusion Criteria**		
**Graft inclusion criteria**	Any of the following:<br>• Donor risk index >2.0 (Feng, 2006)<br>• BAR score greater than 9 (Dutkowski 2012)<br>• Graft macrosteatosis > 30%<br>• Donor warm ischaemic time (defined as the period between the systolic blood pressure less than 50 mmHg to the time of commencing donor aortic perfusion) in DCD donors > 30 minutes,<br>• Peak donor aspartate and alanine transaminases > 1000 IU/ml (AST/ALT)<br>• Anticipated cold ischaemic time > 12 hours for DBD or 8 hours for DCD livers<br>• Suboptimal liver graft perfusion as assessed by a consultant transplant surgeon and documented by graph photography.	
**Graft Exclusion**	• Grafts from patients with active Hepatitis B, C or HIV infection<br>• Livers with cirrhotic macroscopic appearance<br>• Livers with advanced fibrosis<br>• DCD grafts with donor warm ischaemic time (systolic blood pressure less than 50mmHg to aortic perfusion) more than 60 minutes<br>• Excessive cold ischaemic times (DBD more than 16 hours / DCD more than 10 hours)<br>• Paediatric donor (<18 years old)<br>• ABO incompatibility	

**Recipient inclusion criteria**	• Primary adult liver transplant<br>• Low to moderate transplant risk candidate suitable for marginal graft, as assessed by the Liver Unit liver transplant listing multi-disciplinary team meeting.
**Recipient exclusion criteria**	• High risk transplant candidates not suitable for a marginal graft<br>• Patients with complete portal vein thrombosis diagnosed prior to the transplantation<br>• Patients with fulminant hepatic failure<br>• ABO incompatibility<br>• Patient unable to consent<br>• Patients undergoing transplantation of more than one organ<br>• Contraindication to undergo magnetic resonance imaging (e.g. as patients with pacemaker).
**Criteria for transplantation**	• Lactate less than or equal to 2.5mmol/L<br><br>And 2 or more of the following within 4 hours –<br>• Evidence of bile production<br>• pH ≥ 7.30<br>• Metabolism of glucose<br>• HA flow ≥150ml/min and PV flow ≥500ml/min<br>Homogenous perfusion
**Contact Details**	
**Trials office contact details**	**VITTAL Trial Office**<br>D3B Trial Management Team<br>Cancer Research UK Clinical Trials Unit (CRCTU)<br>1st Floor,<br>Open Plan East, ITM,<br>Heritage Building,<br>Mindelsohn Way,<br>Edgbaston, Birmingham,<br>B15 2TH<br><br>**Enquiries**<br>☎ 0121 371 8467<br>🖷 0121 371 8028<br>✉VITTAL@trials.bham.ac.uk

TRIAL SCHEMA

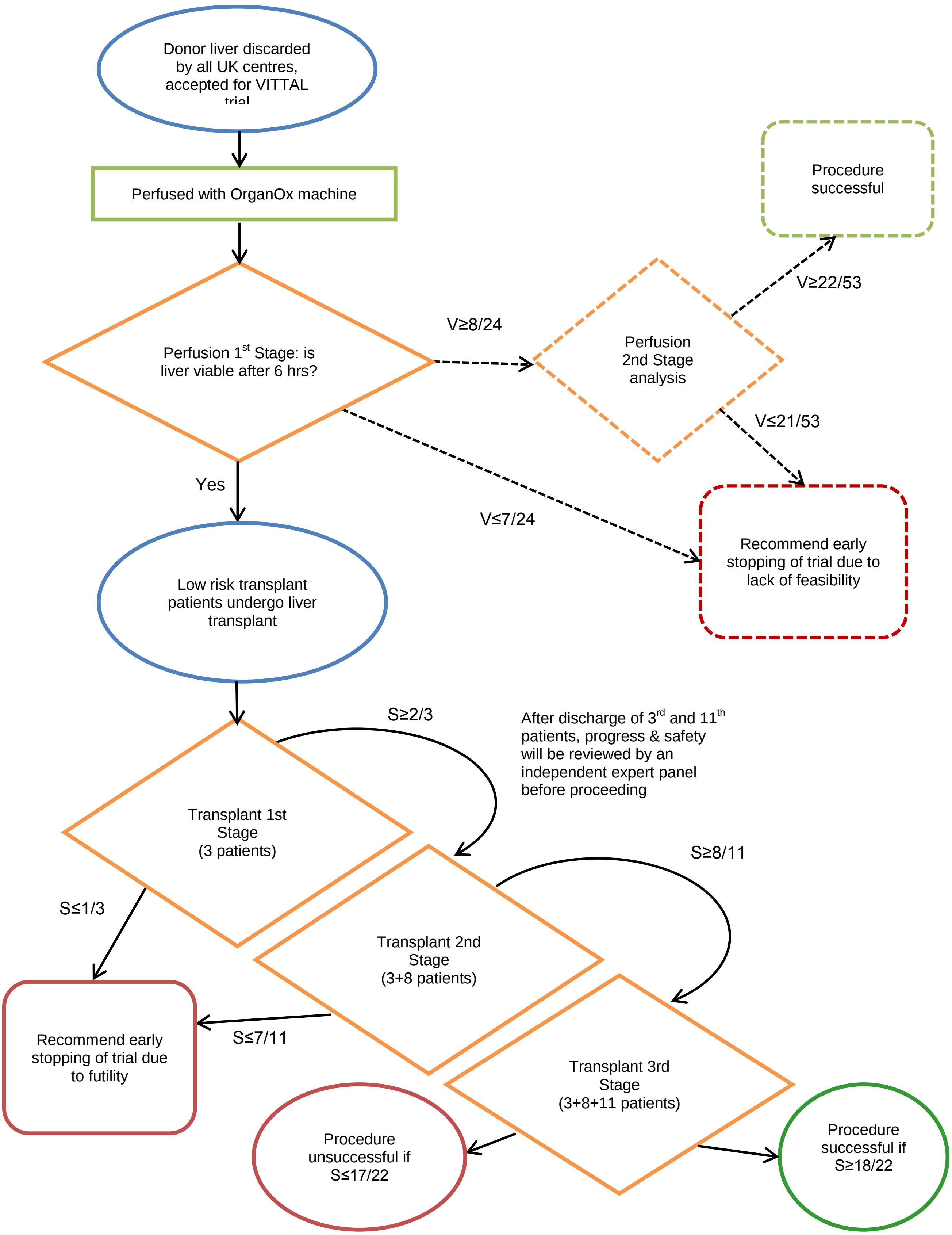

Figure 1 Trial Schema

V = Viable liver S = Successful transplant

SCHEDULE OF EVENTS

Table 1: Liver Schedule of Events

<table>
<tr><td colspan="3">Liver registration and subsequent placing on OrganOx Metra™ Device (4+ hours prior to transplantation)</td></tr>
<tr><td>Donor liver eligibility assessment</td><td colspan="2">X</td></tr>
<tr><td>Liver Biopsy 1 (pre-perfusion at back bench)</td><td colspan="2">X</td></tr>
<tr><td>Attachment to OrganOx metra™ Device</td><td colspan="2">X</td></tr>
<tr><td>OrganOx metra ™ assessment (see criteria in section 4)</td><td colspan="2">X</td></tr>
<tr><td>Liver assessment decision point</td><td>Liver suitable for transplant
Follow Patient Registration table below (Work Package 1 (WP1))</td><td>Liver not suitable for transplant
Liver will be used in Work Package 2 (WP2)</td></tr>
<tr><td>Liver Biopsy 2 (at end of perfusion)</td><td colspan="2">X</td></tr>
</table>

Table 2: Patient Schedule of Events

Patient Registration	**Screening**	**Visit 1 Transplant** Day 0	**Visit 2** Day 30 (+/- 3days)	**Visit 3** Day 90 (+ 3 days)	**Visit 4** Day 180 (+ 30 days)	**Extended follow up** 12 month + 24 month (+/- 30 days)
Informed consent	**X**					
Eligibility assessment	**X**	**X**				
Patient history	**X**	**X**				
Standard routine blood tests*	**X**	**X**	**X**	**X**	**X**	**X**
MELD (automatically calculated)		**X**				
UKELD (automatically calculated)		**X**				
Trial specific additional patient samples blood and urine(see Appendix 6)		**X**	**X**	**X**	**X**	
PBMC Collection (see Laboratory Manual)		**X**	**X**	**X**	**X**	
Liver Biopsy 4 (see table 3)		**X**				
Quality of Life questionnaire (EQ-5D-5L)		**X**	**X**		**X**	
Patient Resource Log at Visit 1 discharge		**X**				
Adverse/ Clinical events	**X**	**X**	**X**	**X**	**X**	**X**
Concomitant medications	**X**	**X**	**X**	**X**	**X**	**X**
MRCP					**X**	

* Standard routine blood tests - Full blood count (FBC), urea, electrolytes, liver function tests (LFTs), AST, GGT, eGFR, international normalised ratio (INR)

Table 3: Time points of liver biopsies

Liver Biopsy 1	Pre-perfusion [menghini liver biopsy and common bile duct biopsy]
Liver Biopsy 2	After 4 hours of perfusion [menghini liver biopsy]
Liver Biopsy 3	At end of perfusion (only taken if perfusion exceeds 6 hours) [menghini liver biopsy]
Liver Biopsy 4	Prior to abdominal closure after re-perfusion [menghini biopsy and common bile duct biopsy]

ABBREVIATIONS

ALT	Alanine transaminase
AST	Aspartate transaminase
ATP	Adenosine tri-phosphate
BMI	Body Mass Index
CIT	Cold Ischaemic Time
CRF	Case Report Form
DBD	Donor/donation after Brain Death
DCD	Donor/donation after Circulatory Death
DRI	Donor Risk Index
EAD	Early Allograft Dysfunction
eCRF	electronic Case Report Form
ESLD	End Stage Liver Disease
FFP	Fresh Frozen Plasma
FWIT	Functional Warm Ischaemic Time
GGT	Gamma-glutyl transferase
HCC	Hepatocellular Carcinoma
HD	Haemodialysis
HDF	Haemodiafiltration
HF	Haemofiltration
HMP	Hypothermic Machine Perfusion
HTK	Histidine-Tryptophan-Ketoglutarate
INR	International Normalised Ratio
ITU	Intensive Therapy Unit
IFU	Information For Use
LFT	Liver Function Test
MAP	Mean Arterial Pressure
MELD	Model For End-Stage Liver Disease
mmHg	mm Mercury (Pressure)
NHSBT	NHS Blood and Transplant
NMLP	Normothermic Machine Liver Perfusion
PBMC	Peripheral blood mononuclear cell
pO_2,	Partial pressure of oxygen
pCO_2	Partial pressure of carbon dioxide
RBC	Red Blood Cells
RRT	Renal Replacement Therapy
SCS	Static Cold Storage
TMG	Trial Management Group
UHBFT	University Hospital Birmingham Foundation Trust

UKELD	UK End Stage Liver Disease score
UW	University of Wisconsin
WP1	Work Package 1
WP2	Work Package 2

TABLE OF CONTENTS

1 Background 1
1.1 Liver transplantation for end-stage liver disease 1
1.2 Epidemiology 1
1.3 The shortage of donor organs 1
1.4 Responding to the shortage 1
1.4.1 Opt-in or opt-out? 1
1.4.2 Living donation 2
1.4.3 The use of “marginal” (or “extended criteria”) donor organs 2
1.5 Reconditioning of donor organs 3
1.5.1 Cold storage of organs 3
1.5.2 In-situ organ reconditioning 4
1.6 Normothermic Machine Liver Perfusion (NMLP) 4
1.6.1 History of NMLP 4
1.6.2 Devices 4
1.6.3 Benefits of NMLP 5
1.7 Research to date 6
1.7.1 Pre-clinical research and pilot study 6
2 Hypothesis, objectives and outcome measures 7
2.1 Hypothesis 7
2.2 Objectives and outcome measures 7
2.2.1 Co-Primary objectives and outcome measures 7
2.2.2 Secondary objectives and associated outcome measures 7
2.2.3 Exploratory objectives and outcome measures 8
3 Trial Design 9
4 Eligibility 10
4.1 Graft Inclusion Criteria 10
4.2 Graft Exclusion Criteria 10
4.3 Criteria for transplantation (OrganOx *metra* ™ assessment) 10
4.4 Recipient Inclusion Criteria 11
4.5 Recipient Exclusion Criteria 11
5 Screening and Consent 12
5.1 Donor Consent 12
5.2 Transplant Recipient Informed Consent 12
6 Trial Entry, Transplant Procedure and Post-transplant Visit Schedule 14
6.1 Stage 1: Liver Registration 15
6.1.1 Times to be recorded for DBD donors are as follows: 15
6.1.2 Times to be recorded for DCD donors are as follows: 16
6.1.3 Donor information 16
6.2 Stage 2: Patient registration and transplant 16
6.2.1 Recipient information 16
6.2.2 Normothermic Machine Liver Perfusion information 17
6.2.3 Operative information 17
6.2.4 Donor demographics 18
6.2.5 OrganOx *metra* ™ Information print-outs 18
6.2.6 Visit 1 – Transplant (Day 0) and Inpatient stay 18

6.3 Post-Transplant Visit Schedule ... 20
6.3.1 Visit 2 - Day 30 post-op (+/- 3 days) ... 20
6.3.2 Visit 3 – 90 days post-op (+3 days) ... 21
6.3.3 Visit 4 – 180 days post-op (+30 days) ... 22
6.4 Extended follow up – 12 month post - transplant (+/- 30 days) and 24 month post – transplant (+/- 30 days) ... 23
6.5 Assessments ... 24
6.5.1 Blood chemistry and haematology as part of standard of care ... 24
6.6 Evaluation of potential biomarkers of graft viability (Work Package 2) ... 25
6.6.1 Histological analysis of tissue samples obtained during NMLP and following transplantation. ... 25
6.6.2 Biochemical analysis and other studies of perfusate samples generated during NMLP and following transplantation. ... 26
6.7 Treatment Compliance ... 26
6.8 Supportive Treatment ... 26
6.9 Concomitant Medication ... 26
6.10 Health economic analysis ... 26
6.11 Patient Retention and Withdrawal ... 27
7 Intervention Details ... 27
7.1 Device Description- OrganOx *metra* ™ ... 27
7.2 Manufacturing Organisation ... 28
7.3 The OrganOx *metra* ™ Base Unit ... 28
7.3.1 Disposable set ... 28
7.3.2 Perfusion solutions ... 29
7.4 Device safety ... 29
7.5 Device labelling ... 30
7.6 Device accountability ... 30
7.7 Device maintenance ... 30
7.8 Device logistics ... 30
7.8.1 Logistical considerations ... 30
7.8.2 Logistical arrangements ... 30
8 Adverse Event Reporting ... 31
8.1 Reporting Requirements ... 31
8.1.1 Adverse Events, Device Deficiencies and Adverse Device Effects ... 31
8.1.2 Known liver transplant post-operation complications ... 32
8.1.3 Serious Adverse Events and Serious Adverse Device Effect ... 32
8.1.4 Events that do not require expedited reporting ... 33
8.1.5 Events that do not require reporting on a Serious Adverse Event Form ... 33
8.1.6 Provision of follow-up information ... 34
8.1.7 Trials Office ... 34
8.2 Reporting to the Competent Authority and main Research Ethics Committee ... 34
8.2.1 Adverse Events and Adverse Device Effects ... 34
8.2.2 Serious Adverse Device Effect ... 34
8.2.3 Serious Adverse Events ... 34
8.2.4 Unanticipated Serious Adverse Device Effects ... 34
8.2.5 Other safety issues identified during the course of the trial ... 34
8.2.6 Investigators ... 35
8.2.7 Reporting period ... 35

8.2.8 Data Monitoring Committee 35
8.3 Reporting Procedure 35
8.3.1 Provision of follow-up information........ 35
8.3.2 Trials Office........ 35
9 Data Handling and Record Keeping 36
9.1 Data Collection 36
10 Archiving........ 37
11 Quality Management 37
11.1 Site Set-up and Initiation 37
11.2 On-site Monitoring 37
11.3 Central Monitoring 38
11.4 Audit and Inspection 38
11.5 Notification of Serious Breaches 38
12 End of Trial Definition 38
13 Statistical Considerations 39
13.1 Determination of Sample Size 39
13.2 Definition of Objectives and Outcome Measures 40
13.3 Analysis of Outcome Measures........ 40
13.3.1 Primary Analysis 40
13.3.2 Secondary Analysis 41
13.3.3 Exploratory Analysis 41
13.3.4 Health Economic Analysis 41
13.4 Planned Sub Group Analyses 41
13.5 Planned Interim Analysis 41
13.6 Planned Final Analyses 42
14 Trial Organisational Structure........ 42
14.1 Sponsor 42
14.2 Coordinating Centre 43
14.3 Trial Management Group........ 43
14.4 Trial Steering Committee........ 43
14.5 Data Monitoring Committee........ 43
15 Finance........ 44
16 Ethical and Regulatory Considerations........ 44
17 Confidentiality and Data Protection........ 45
18 Insurance and Indemnity 45
19 Publication Policy 45
20 Reference List........ 46
Appendix 1 – CLAVIEN-DINDO CLASSIFICATION OF SURGICAL COMPLICATIONS 48
Appendix 2 – EQ-5D-5L........ 49
Appendix 3 – LIST OF UK TRANSPLANT CENTRES 52
Appendix 4 – DONOR RISK INDEX 52
Appendix 5 – BAR SCORE........ 52
Appendix 6 – BIOLOGICAL SPECIMENS........ 53
Appendix 7 – DEFINITION OF ADVERSE EVENTS 57

Appendix 8 – WMA DECLARATION OF HELSINKI 59

INDEX OF TABLES AND FIGURES

Table 1: Liver Schedule of Events viii
Table 2: Patient Schedule of Events ix
Table 3: Time points of liver biopsies x
Table 4 List of known complications 32
Table 5 Case Report Forms (CRFs) 36

Figure 1 Trial Schema vii
Figure 2 Criteria for donor quality as per British Transplantation Society UK Guidelines for Donors after Circulatory death 3
Figure 3 Individual patient and donor liver pathway 14
Figure 4: OrganOx *metra* ™ Machine 28

1 BACKGROUND

1.1 Liver transplantation for end-stage liver disease

Liver transplantation is a highly successful treatment of end stage liver disease, fulminant hepatic failure and early stage primary liver cancer. Deaths from liver disease have soared by 40 per cent in a decade and continue to rise. Liver disease kills 11,000 a year in England and the average age of death from liver disease (59 years), continues to fall [1]. In contrast to the dialysis offered to patients with end-stage kidney disease, as yet there is no technology or treatment available to support patients with liver failure for an extended period. Over the past 50 years, transplant techniques and outcomes have greatly improved and 5-year survival rates of 70-80% mean transplantation has become the mainstay of treatment for an increasing number of patients with chronic liver disease, metabolic disorders, acute liver failure and malignancy. As such, the demand for donor livers greatly exceeds supply and approximately 20% of patients die whilst awaiting transplantation [2].

1.2 Epidemiology

In Europe the most common indications for liver transplantation are cirrhosis (68%), malignancy (14%), and acute hepatic failure (8%). The main causes for cirrhosis in Europe are the hepatotropic viruses (A, B and C) and alcoholic liver disease[3]. Non-alcoholic fatty liver disease is an emergent cause and the incidence continues to rise despite health campaigns. In the UK it is predicted that HCV-related cirrhosis and deaths from hepatocellular carcinoma (HCC) will increase substantially during the next decade. As a result, the demand for suitable donor livers will continue to rise exacerbating the existing shortage.

1.3 The shortage of donor organs

Between March 2014 and April 2015, 1206 patients joined the liver transplant waiting list in the UK and for the 3rd time in a decade the number of transplants carried out reduced (842) [4]. This shortfall is reflected across other countries to the extent that a patient is now more likely to die within the first 12 months of being listed than the first 12 months post-transplant. Over the past decade there has been a very modest increase in the use of standard or 'ideal' organ donors (those retrieved following a diagnosis of brain-stem death, DBD) and so to respond to this deficit, centres have utilised donors following circulatory death (DCD) and sub-optimal "marginal" donors (those of older age, livers with a presence of steatosis etc.).

1.4 Responding to the shortage

1.4.1 Opt-in or opt-out?

There is a lack of well-controlled scientific evidence on which to base decisions regarding policy-making and opinions are strong and divided. Spain has the highest organ donation rates and operates an opt-out system; however the rise in rates only started approximately 10 years after the introduction of the opt-out system. Wales is the most recent country to go down this route, however unlike in Spain, next of kin consent is still required before patients can be made organ donors. More likely, the increased Spanish donation rates are due to the

creation of a transplant coordination network that operates at hospital, regional and national levels, the placement of transplant coordinators at each procurement hospital, and the improvement in the quality of information received by the general public.

1.4.2 Living donation

Living donation is one potential means to increase the number of liver transplants, using surgical techniques developed for 'liver splitting' which normally uses a single liver for transplantation into two recipients. The major limitations are most patients do not have a willing/suitable living donor and there are concerns about the risks to the healthy donor. The reported risk of donor death is estimated at 0.2% but the risk of serious complications is much higher [5, 6]. Living donor transplantation will be unlikely to have a significant impact on the shortage of donor livers in most countries.

1.4.3 The use of "marginal" (or "extended criteria") donor organs

A rising proportion of transplants are carried out using "marginal" or "extended criteria" grafts, procured from obese or elderly donors with multiple co-morbidities [7]. These livers are significantly more susceptible to cold storage-related ischaemic injury, which increases the risk of graft failure and recipient morbidity and mortality. Despite the use of these sub-optimal grafts, in 2014/15, of 1282 solid organ donors, only 924 (72.1%) livers were deemed suitable for retrieval and 812 (63.3%) were subsequently transplanted NHS Blood and Transplant (NHSBT) Activity Report 2014/15). During a DBD retrieval, although there are physiological changes as a result of brain death, there is no functional warm ischaemic time (FWIT) as the donor's heart continues to beat and oxygenation is maintained until the organ is perfused with chilled perfusate. During retrieval of a DCD organ, the FWIT starts when the systolic blood pressure has a sustained (i.e. at least 2 min) fall below 50 mmHg (or the haemoglobin oxygen saturation falls below 70%) and extends up to the onset of cold *in-situ* perfusion [8]. The duration of the FWIT is the important determinant of outcome and the recent document 'Donation After Circulatory Death' published by a steering group on behalf of the British Transplantation Society and Intensive Care Society suggested that the stand-down time from the onset of functional warm ischaemia for DCD liver transplantation was 30 minutes (although 20 minutes is ideal), and that age was an important factor. Because of this, a number of livers will be retrieved from DCDs that fall into the "marginal donor" category and may not go on to be transplanted.

Several donor parameters have been identified as relative risk factors for poor outcome, including age; steatosis; DCD donation; split livers; prolonged cold ischaemia time (>12 hours). These were all developed using North American data and formulated into an algorithm known as the Donor Risk Index (DRI), and later validated using European data [9, 10]. The British Transplantation Society have published their own guidelines on the utilisation donor organs and use criteria in Figure 2 to distinguish between grafts of varying quality.

Criteria for donor quality as per British Transplantation Society UK Guidelines for Donors after Circulatory death

Good livers – *All should be used* (DBDs and DCDs)	Ideal Livers – *All should be used* (DCDs)	Marginal Donors – *Use selectively* (DCDs)	Absolute contraindications to using liver as donor organ
Age <50<br>Normal LFTs<br><5 days on ITU<br>Low levels of inotropic support<br><30% Steatosis<br>No active sepsis	Age <50 years<br>Weight <100kg<br>FWIT <20 mins<br>CIT <8 hours<br><15% Steatosis<br>ICU stay <5 days	Age >50 years<br>Weight >100kg<br>FWIT 20-30mins<br>CIT 8-12 hrs<br>>15% Steatosis<br>ICU stay >5 days	Steatosis >30%<br>ESLD<br>Acute liver failure<br>Acute liver injury that's not improving

Figure 2 Criteria for donor quality as per British Transplantation Society UK Guidelines for Donors after Circulatory death

1.5 Reconditioning of donor organs

1.5.1 Cold storage of organs

The current standard of donor liver preservation is based on static cold storage (SCS) [11]. During static cold storage, organs are flushed and cooled with specific chilled preservation solutions (University of Wisconsin [UW] solution is used most commonly although Histidine-Tryptophan-Ketoglutarate (HTK) solution is also widely used) and ice is added to the abdominal cavity. After retrieval, the organ is placed in fluid-filled sterile plastic bags for transportation and stored in an ice-box in preservation solution until transplantation. Although the available preservation solutions differ in chemical composition, the function is essentially the same. The hypothermia aims to reduce the liver's metabolic activity and the solution aims to reduce the cellular swelling. This is a consequence of anaerobic metabolism resulting in depletion of adenosine tri-phosphate (ATP) stores leading to influx of free calcium and activation of phospholipases. Cooling the organ slows metabolism approximately 12-fold but cannot prevent its dysfunction and the eventual destruction of cellular integrity. Ischaemia-reperfusion is an important factor influencing graft outcome. The ischaemic phase starts early in the procurement process (swings in blood pressure following brain-death or due to the functional warm ischaemic time in non-heart beating donors) and triggers a complex cascade of cellular and molecular events including the release of pro-inflammatory mediators and chemotaxis of cell types that initiate progressive immunological processes. During the reperfusion phase, "the reflow paradox" causes infiltration of the tissues by leucocytes and cellular injury occurs through a series of pathways that include lipid peroxidation and the creation of reactive oxygen species. The most common manifestation of the ischaemia-reperfusion process is delayed graft function , which is the inability of the organ to fulfil the physiological needs of the recipient and is associated with graft failure, re-transplantation and death. Static cold storage therefore is a) unable to reverse the injury sustained during donor death and procurement, b) causes injury due to the cooling process, c) limits the preservation time and d) prevents physiological assessment prior to transplantation.

1.5.2 In-situ organ reconditioning

In an attempt to reverse or diminish the injury, many cytoprotective strategies have been tested in experimental models of transplantation and several have been shown to have therapeutic potential, including various antioxidants, inflammation inhibitors, vasodilating agents, inhibitors of chemotaxis or neutrophil infiltration. Treatment of the organ during preservation has major logistic and ethical advantages over any attempt to achieve the same effects by treating the donor (therapeutic interventions before declaration of death are not currently permitted unless they are of potential benefit to the donor). Recently there has been published early experience with normothermic regional perfusion of DCD donors, nevertheless the feasibility and benefit of this experimental approach is yet to be shown [12].

1.6 Normothermic Machine Liver Perfusion (NMLP)

1.6.1 History of NMLP

Bretschneider and Starzl first attempted machine perfusion of the liver in the late 1960's. Although hypothermic machine perfusion (HMP) has been investigated in an experimental setting and shown some promise in clinical studies, normothermic machine liver perfusion (NMLP) combats the limitations of SCS previously described by aiming to maintain the organ at the body's natural temperature whilst providing oxygen, nutrition and the essential substrates necessary for adequate cellular metabolism. Providing a homeostatic environment theoretically enables us to extend our storage period and test the organs physiological parameters. To date only one clinical trial of 20 adult recipients of livers maintained by HMP has been published showing a reduction in early graft dysfunction (5% vs 25% $p<0.08$) as well as a significant reduction in serum injury markers in the HMP group. A joint pilot trial between Oxford University, King's College Hospital London and University Hospitals Birmingham Foundation Trust (UHBFT) recruited 20 patients into a phase 1 study and concluded the procedure was feasible and safe when used on current conventional donor acceptance criteria [13]. In addition, a 220 patient phase III international clinical trial entitled "COPE WP2", is currently being performed and has completed recruitment. The UHBFT Liver Unit contributed to this multi-centre international trial by randomising 50% of the study patients.

1.6.2 Devices

NMLP permits a period within which the donor organ can be functionally assessed thereby increasing transplant safety. It can also extend organ preservation times to improve transplant logistics and donor organ utilisation. There are several devices available on the market, but only the OrganOx *metra* ™ has been widely used in the clinical transplant setting [13]. Our team has performed over 70 liver transplant with grafts preserved on this machine and has gained broad experience by using this device.

There are two other companies producing machines for NMLP. Organ Assist developed the Liver Assist device, a non-transportable, non-automated device requiring considerable technical support and attention. Transmedics is testing Organ Care System Liver machine, but that does not have in-built viability assessment, is only suitable for a maximum of 12 hours preservation and has not yet been tested in clinical setting. The OrganOx *metra* ™ is the leading device in terms of the number of clinical transplants undertaken, with more than 100 machine-perfused livers transplanted in the Phase III randomised European trial, together with 20 livers in the Phase I safety study and further on-going trials in North America.

Competing products have either performed limited or no numbers of clinical transplants and with respect to the additional, above-mentioned limitations, we have decided to use the OrganOx *metra* ™ device for the proposed study.
The device consists of a unit that cradles the liver, a perfusate reservoir, oxygenators, pumps operating physiological pressures and tubing systems to connect the unit to the portal vein and the hepatic artery. The constituents of the perfusate can vary but generally consist of whole blood for oxygen carriage, sources of nutrition (glucose, insulin, amino acids), anti-thrombotic agents (heparin, epoprostenol), antibiotics and acid-base agents which help reduce cellular oedema, cholestasis, microvascular injury and the effects of free-radicals .

1.6.3 Benefits of NMLP

NMLP does not simply benefit marginal DCD organs that have been exposed to a damaging FWIT. Brain-stem death is a catastrophic physiological event associated with profound hypotension (parasympathetic response) followed by hypertension, tachycardia and high levels of circulating catecholamines (sympathetic surge) followed by another reduction in the sympathetic outflow. These dramatic swings can cause significant graft ischaemia prior to retrieval. Diabetes insipidus occurs in 70-80% of brain dead patients causing severe hypernatraemia (associated with primary liver graft non-function), hypokalaemia, hypocalcaemia, hypophosphataemia and hypomagnesaemia. Pirenne *et* al described seven cases when livers from DBD's between 70 and 80 years old were used with "favourable outcomes". NMLP could play an important role in preconditioning and assessing such organs prior to transplantation.

Hellinger et al were unable to identify a benefit using NMLP however in 1997 it was the first study of its kind. In 2002, Schon used NMLP to preserve and re-condition livers that had been exposed to 1 hour of warm ischaemia. These livers were then transplanted into pigs which all survived longer than 7 days. The group that received livers preserved using SCS had no survivors. Several studies have been published by the Oxford group, responsible for OrganOx *metra* ™. Imber et al published results from a study on a porcine model comparing NMLP with SCS controls. They showed livers preserved using NMLP were significantly superior ($P<0.05$) to SCS livers in terms of bile production, factor V production, glucose metabolism, and galactose clearance, whilst SCS livers showed significantly higher levels of hepatocellular enzymes in the perfusate and exhibited more cellular damage. The same year they successfully perfused and maintained 5 porcine livers for 72 hours, managing to maintain normal physiological parameters, pH, protein synthesis and histological architecture. In 2009, Brockman et al simulated DBD and DCD scenarios in a porcine model. After five hours of preservation (NMLP vs SCS) there was no difference seen in preservation method in either the DCD or DBD graft recipients. After 20 hours of preservation however, grafts (DCD and DBD) that had been preserved using NMLP were superior to their SCS counterparts with respect to enzyme release, histological changes and recipient survival. Of note there was no difference in survival between DCD and DBD NMLP-preserved graft recipients (83% and 86% respectively).

1.7 Research to date

1.7.1 Pre-clinical research and pilot study

Our team's pre-clinical research on rejected human livers has demonstrated that presence of bile production, in combination with maintenance of physiological pH, metabolism of lactate and stable blood flow rates, are sensitive parameters predictive of organ viability. In April 2014, the UHBFT Novel Therapeutics Committee approved a pilot clinical project for transplantation of five reconditioned liver grafts, initially deemed unusable for transplantation. In this series, livers were declined by all the UK transplant units, after which NMLP commenced following a variable period of static cold storage. Still, five out of six tested livers met the viability criteria and were successfully transplanted [14]. Although this pilot project showed that viability testing could transform the organ selection and acceptance process of high-risk livers, our observation provided primarily the feasibility and short-term outcome data. Six months follow up of the last included transplant in this series, in combination with normal Liver Function Tests (LFTs) in all included patients is likely to exclude any early clinically relevant form of ischaemic type biliary complication, one of the main problems in recipients of DCD livers. The histology of the post-NMLP common bile duct biopsy is also not suspicious for the development of ischaemic bile duct lesions with less than 50% epithelial necrosis of deep peri-biliary glands, no thrombi and minimal arteriolar necrosis. In addition, our study shows the feasibility to perform NMLP following SCS and inspection at the transplant centre, with logistical and financial advantages, and may allow targeting livers that would benefit most from NMLP. More research in this area is required and this was recognised by the Health Innovation Challenge committee of the Wellcome Trust who have recently awarded our study group a research grant to set up and run this trial. We have demonstrated so far, that a substantial proportion of currently rejected liver allografts might be salvaged by subjecting them to NMLP and viability testing. Use of this technology can transform the utilisation of high-risk organs and may improve access to treatment for thousands of patients awaiting liver transplantation globally.

2 HYPOTHESIS, OBJECTIVES AND OUTCOME MEASURES

2.1 Hypothesis

NMLP enables clinicians to objectively assess liver function during the perfusion process and using defined criteria, one can determine whether a rejected liver is viable and can be safely used for transplantation.

2.2 Objectives and outcome measures

2.2.1 Co-Primary objectives and outcome measures

Objective 1a - Establish the feasibility of NMLP as a means to increase the number of transplantable livers.

Outcome measure 1a – "Rescue rate" i.e. the proportion of rejected livers that can be used for transplantation having been deemed viable following a period of machine perfusion. As per study design, NMLP is feasible if ≥22/53 perfusions of rejected liver grafts result in transplantation.

Objective 1b – Achievement of successful transplantation using rejected liver grafts

Outcome Measure 1b – 90-day patient survival (achievement of successful transplantation of previously rejected donor liver following viability testing using NMLP.

2.2.2 Secondary objectives and associated outcome measures

1. Assess the liver graft function following transplantation (by incidence of primary non-function, and early allograft dysfunction):
   - Liver function tests
   - 90-day graft survival
   - 12-month patient and graft survival

   The secondary endpoints and other outcome measures will be compared with a contemporary matched recipient group using the following matching criteria:
   - Patient Characteristics: Age, Sex, BMI, MELD, UKELD, Aetiology
   - Donor Liver Characteristics: DCD or DBD, Sex

2. Assess morbidity associated with receipt of extended criteria graft that had previously been rejected:
   - Adverse event rates and severity, graded according to the Clavien-Dindo classification [15] (Appendix 1)
   - Requirement of renal replacement therapy
   - Incidence of biliary complications
   - Incidence of vascular complications
   - Biopsy-proven acute rejection
   - Reoperation rate
   - Length of intensive therapy unit stay
   - Length of hospital stay

3. Assess the physiological response to reperfusion of the perfused grafts:
   - Post-reperfusion syndrome (Defined as a decrease in mean arterial pressure (MAP) of more than 30% from the baseline value for more than one minute during the first five minutes after reperfusion (assessed in the context of inotrope use))

4. Identify impact upon quality of life after transplantation with these liver grafts
   - Quality of life by delivery of the EQ-5D-5L questionnaire (Appendix 2) at baseline, day 30 and 6 months post-transplant.

2.2.3 Exploratory objectives and outcome measures

1. Identify novel biomarkers that are indicative of liver quality and function (WP2):
   - This will be performed through careful analysis of biological samples and through correlation of findings with perfusion outcome and if transplanted, clinical outcome. Tissue samples will be analysed to identify histological evidence of reperfusion injury in post-reperfusion biopsies (taken immediately prior to abdominal closure). These will be compared to baseline pre-reperfusion biopsies (on removal of the liver from SCS/NMLP) and graded

2. To assess the health economic implications of using this technique to determine organ viability:
   - Logistical costs, measured using national unit costs where available.
   - Healthcare resource use; measured by a combination of hospital episode records and a patient-completed resource use log.

3 TRIAL DESIGN

The proposed trial design is based on exactly defined donor organ selection and recipient criteria. The primary aims of the clinical trial are:

(A) Use of NMLP to identify the proportion of transplantable liver grafts from the currently rejected donor organ pool. This NMLP use will quantify the number of potentially salvageable livers and the benefit this approach may bring to patients awaiting liver transplantation in the UK.
(B) Achievement of successful transplantation as measured by 90-day patient survival (a nationally accepted, monitored and continuously audited outcome following liver transplantation)

The proposed design is a non-randomised, prospective, single arm trial, performing normothermic machine liver perfusion (NMLP) on rejected livers until it reaches 22 clinical transplants. It is designed with two linked components to assess (A) the feasibility of NMLP in rejected organ recovery using an established 2-stage design [16] and (B) successful transplantation using an optimal 3-stage design [17].

The statistical calculation powered the trial with a particular focus on recipient safety, but also on financial feasibility of NMLP implementation to the organ selection and recovery pathway. Under (A), it is anticipated that NMLP will achieve an organ recovery rate of at least 50%, with an undesirable rate of 30% or less as it will not be economically feasible. Using a Simon's two-stage design (1989), with an interim assessment after 24 livers (requires ≥8 recovered), a sample size of up to 53 livers undergoing NMLP might be required, with alpha = 0.05 and beta =0.1. NMLP will be considered both clinically and economically feasible for organ recovery if there are at least 22 recovered livers (out of 53 perfused livers).

The mean 90-day survival rate for "standard" liver transplants is 93% (Annual Report on Liver Transplantation, NHS Blood and Transplant, 2014) [18]. Under (B) for viable marginal livers following NMLP, the desirable and undesirable 90-day overall survival rates are 88% and 73% (15% lower) respectively. Using an optimal three stage design (Chen, 1997) with two interim assessments after 3 patients (requires ≥2 successes) and 11 patients (≥8 successes), a sample size of up to 22 patients will be required, with alpha and beta = 0.2. The procedure will be considered successful if there are at least 18 successes (out of 22).

4 ELIGIBILITY

This clinical trial involves the use of rejected donor liver graft and transplant recipients. Below are listed the inclusion and exclusion criteria for the liver graft and recipient respectively.

4.1 Graft Inclusion Criteria

Donor liver graft must meet *all* of the following inclusion criteria to be considered / eligible for inclusion in the VITTAL trial:

1. Liver from a donor primary accepted with the intention for a clinical transplantation
2. Liver graft was rejected by all the other UK transplant centres via normal or fast-track sequence (see Appendix 3 for list of UK centres)
3. One of the following parameters capturing the objectivity of the liver high-risk status
   - Donor risk index greater than 2.0 [9] (Appendix 4)
   - Graft steatosis greater than 30%
   - BAR score greater than 9; [19] (Appendix 5)
   - Donor warm ischaemic time greater than 30 minutes
   - Anticipated cold ischaemic time greater than 12 hours for DBD or 8 hours for DCD liver grafts
   - Suboptimal liver graft perfusion documented by a photo of macroscopic appearance
   - Liver transaminases (ALT or AST) above 1000 IU/mL

4.2 Graft Exclusion Criteria

Livers meeting any of the following criteria would not be suitable for the VITTAL trial:

1. Grafts from patients with active Hepatitis B, C or HIV infection
2. Livers with cirrhotic macroscopic appearance
3. Livers with advanced fibrosis
4. DCD grafts with donor warm ischaemic time (systolic blood pressure less than 50mmHg to aortic perfusion) more than 60 minutes
5. Excessive cold ischaemic times (DBD more than 16 hours / DCD more than 10 hours)
6. Paediatric donor
7. ABO incompatibility

4.3 Criteria for transplantation (OrganOx *metra* ™ assessment)

To be eligible for use in transplantation perfused livers must

- Reduce the perfusate lactate level to less than or equal to 2.5mmol/L within 4 hours of the start of the perfusion

And meet at least 2 of the following criteria within 4 hours of the start of perfusion:

- Evidence of bile production
- pH greater than or equal to 7.30
- Metabolism of glucose
- Stable arterial flow of more than or equal to 150 mL/ minute and portal flow more than or equal to 500 mL/minute
- Homogeneous graft perfusion with soft consistency of the parenchyma

4.4 Recipient Inclusion Criteria

Subjects must meet *all* of the following inclusion criteria to be eligible for participation in the VITTAL trial:

1. Adult primary liver transplant recipient
2. Patient listed electively for transplantation
3. Low to moderate transplant risk candidate, suitable for marginal graft, as assessed by the UHB liver transplant listing MDT meeting (these are usually candidates with low UKELD score, without cardiovascular comorbidities, with good functional and nutrition status, with patent portal vein and with no history of previous major upper abdominal surgery, e.g. patients transplanted for liver cancer).

4.5 Recipient Exclusion Criteria

Subjects who meet *any* of the following exclusion criteria are excluded from participating in the VITTAL trial:

1. High-risk patients and recipients not suitable for a marginal graft (these are mainly patients with high UKELD score (>62 as per the NHSBT LAG criteria for graft sharing in high risks recipients in the North East of the UK with cardiovascular comorbidities or renal insufficiency, with poor nutrition and performance status or history of major upper abdominal surgery, e.g. patients listed for liver re-transplantation) [http://www.odt.nhs.uk/ search "Liver Allocation Policy"]
2. Patients with complete portal vein thrombosis diagnosed prior to the transplantation
3. Liver re-transplantation
4. Patients with fulminant hepatic failure
5. Patients undergoing transplantation of more than one organ
6. Contraindication to magnetic resonance imaging (i.e. pacemaker fitted)

5 SCREENING AND CONSENT

This clinical trial involves the use of organs from deceased individuals that have been donated for liver transplant through the organ retrieval network managed and coordinated by the NHSBT. Only organs retrieved through this service will be used during this study. Standard NHS donor organ consent, retrieval and allocation process will be utilised.

5.1 Donor Consent

Explicit consent from the donor family is not required for participation in the study as the donation process is unaltered by participation in the trial (no intervention occurs prior to donation), and the intervention does not affect the suitability of the donor organ for transplant. The standard donor organ NHSBT consent procedure will be followed and unless the donor family specifically objects to the organs to be used for clinical research, the consent for organ donation allows donor livers to be considered for the VITTAL trial. During the course of the study, donor details will be kept anonymous (specific study identification codes will be used for each study donor). Donor data will only be made available to authorised staff of the study sponsor, its authorised representatives and regulatory authorities. Anonymised donor data will be used in future publications arising from the study.

5.2 Transplant Recipient Informed Consent

The emergency nature of liver transplantation means that once a potential recipient is called in for a transplant there will be a limited time frame for the consent and screening process to occur. This may not allow sufficient time for the potential recipient to consider the implications of participating in the study. The Liver Unit at UHBFT has an established machine perfusion transplant programme and the NMLP technique explanation, its implication for organ selection and transplant logistics have become a standard part of the transplant assessment process. For this reason, all patients who fulfil the inclusion criteria and come for the phase two transplant assessment process will be given the VITTAL trial Patient Information Sheet. This will provide those patients who are suitable to participate in the VITTAL trial ample time (at least 24 hours) to consider their involvement into the study and to discuss their participation with others outside of the site research team. The VITTAL trial consent can be taken once the potential patient is actively put on the liver transplant waiting list. The patient must be given an opportunity to ask questions which should be answered to their satisfaction. The right of the patient to refuse to participate in the trial without giving a reason must be respected.

If the patient expresses interest to participate in the study, by formally contacting a member of the study team either by telephone, letter or in person, study consent will be obtained by either

1. Verbal consent (verbal consent to be documented fully in the patient medical record – within 12 hours of verbal consent being taken). This written documentation should include the date and version of the Patient Information Sheet given to the patient.
2. Full written consent will be obtained during a face to face meeting by one of the transplant team members during inpatient admission or outpatient appointment. Due to the nature of the study, consent can be obtained immediately prior to / on the day of the liver transplant operation.

It is the responsibility of the Principal Investigator or delegate as captured on the site delegation log to obtain written informed consent for each patient prior to performing any trial related procedure.

A Patient Information Sheet is provided to facilitate this process. Investigators must ensure that they adequately explain the aim, trial treatment, anticipated benefits and potential hazards of taking part in the trial to the patient. The Investigator should also stress that the patient is completely free to refuse to take part or withdraw from the trial at any time.

Written informed consent will be documented by means of a dated signature from the participant and dated signature from the person who presented and obtained the informed consent. The person who obtains consent must be:

1. Suitably qualified and capable of providing information about the study;
2. Capable of answering questions about the study or ensuring that such questions are answered by a suitably qualified individual;
3. Authorised to do so by the local participating centre Principal Investigator – detailed on site delegation log

A copy of the signed Patient Information Sheet and the signed and dated Informed Consent Form will be given to the participant. The original signed form will be retained at the study site and a copy will be placed and/or electronically scanned in the medical notes.
Once the patient is entered into the trial (receives a liver transplant) the patient's trial number should be entered on the Informed Consent Form maintained in the ISF.

Details of all informed consent discussions (initial contact, verbal consent (if applicable) and written consent) should be recorded in the patient's medical notes (within 24 hours of the event – except verbal consent which should be within 12 hours). This should include date of, and information regarding the initial discussion, the date consent was given, with the name of the trial and the version number of the Patient Information Sheet and Informed Consent Form.

Throughout the trial the patient should have the opportunity to ask questions about the trial and any new information that may be relevant to the patient's continued participation should be shared with them in a timely manner. On occasion it may be necessary to re-consent the patient in which case the process above should be followed and the patient's right to withdraw from the trial respected.

Electronic copies of the Patient Information Sheet and Informed Consent Form are available from the Trials Office and should be printed or photocopied onto the headed paper of the local institution.

Details of all patients approached about the trial should be recorded on the Patient Screening/Enrolment Log and with the patient's prior consent, their General Practitioner (GP) should also be informed that they are taking part in the trial. A VITTAL GP Letter is provided electronically for this purpose and should be printed or photocopied onto local headed paper.

6 TRIAL ENTRY, TRANSPLANT PROCEDURE AND POST-TRANSPLANT VISIT SCHEDULE

Due to the study design there are two forms of registration into this study as outlined in the patient and donor pathway (figure 3):

- Stage 1: Liver registration
- Stage 2: Patient registration and transplant

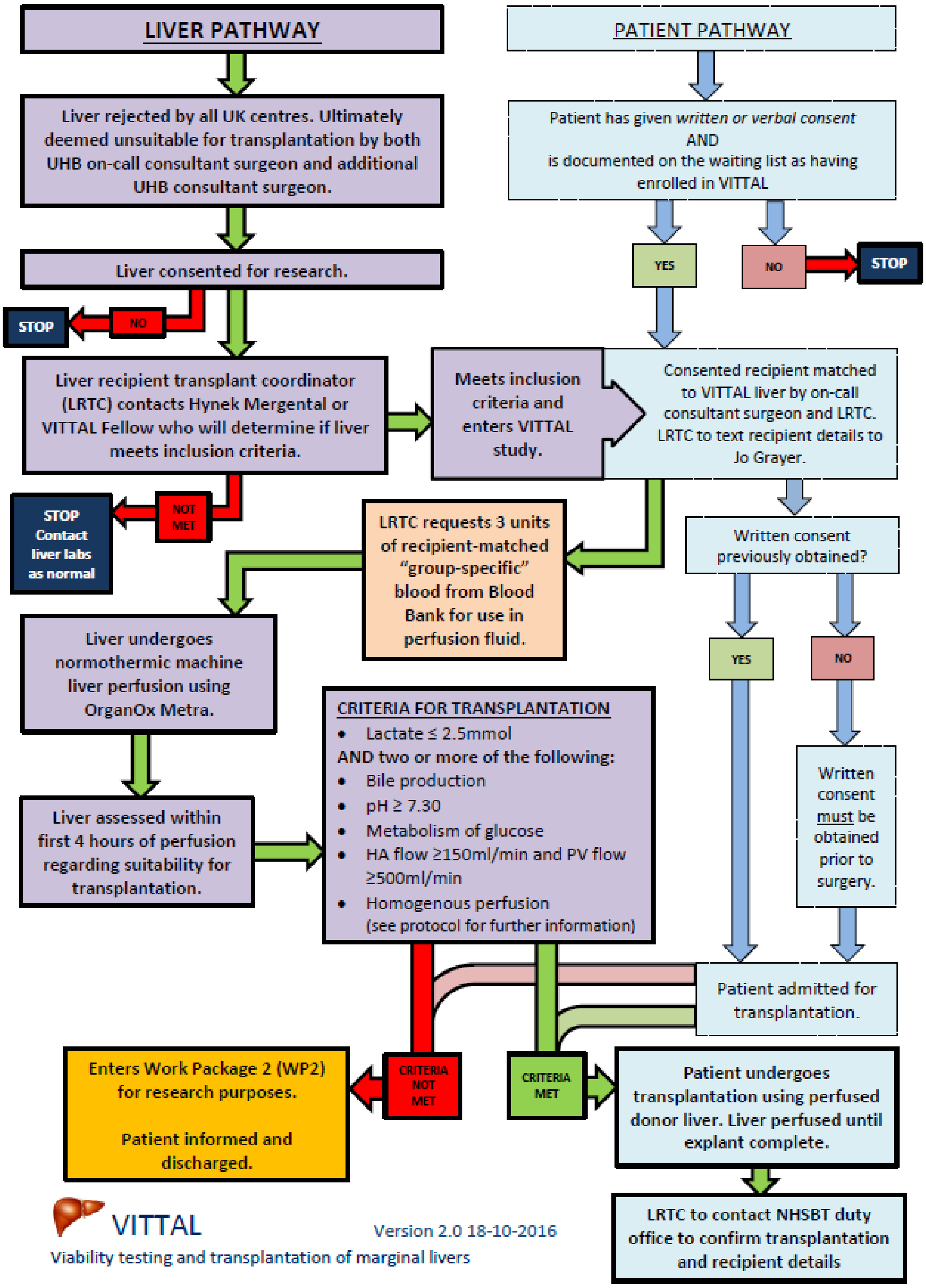

Figure 3 Individual patient and donor liver pathway

This trial will use the OrganOx *metra* ™ device post organ preservation (cold storage) to evaluate organ viability pre-transplant procedure. The OrganOx *metra* ™ device currently has a CE mark for liver organ transport and not evaluation. The use of the device within this clinical trial will be off registration and therefore UK Competent authority (MHRA) clinical trial authorisation will be required.

If the donor liver is suitable for inclusion into VITTAL, arrangements will be made to transport the liver promptly to UHB on static cold storage. The research fellow will contact blood bank to arrange for 3 units of matched recipient-type red blood cells to be cross-matched at UHB for use in the OrganOx *metra*™ device. Ideally, blood will be matched against a valid group and save sample or if not available and if the logistics allow, the patient will be brought into hospital and a group and save sample will be sent to permit cross matching. If the patient lives far away and the liver needs to be perfused before they will arrive, blood will be matched against the last available sample and a repeat sample will be sent on the patients arrival. If the patient is found to have developed new antibodies since the last sample, a discussion between the consultant haematologist and consultant surgeon will take place to discuss the use of the liver. If for some reason the recipient needs to be changed after the perfusion has commenced, blood bank will be asked to check that there is no cross-reactivity between the blood being used on the device, and the new recipient's cross match sample that is sent on their admission.

Following the routine retrieval procedure at the donor hospital the liver will be placed in ice-cold perfusion solution (according to local protocol) on the back-table, and transported. On receipt at UHB, the liver will be assessed and if suitable for inclusion, a suitable recipient will be contacted to come in to hospital. The liver will be prepared according to the procedure for preparing the device for use and placing the organ on the device (described in detail in the OrganOx *metra*™ Instructions for Use (IFU) document (version 13.0, 12-Mar-2016). If the liver meets the criteria for transplantation, the recipient explantation will commence and the procedure for removing the liver from the device is also described in the IFU. Implantation and reperfusion of the liver will proceed as per the usual practice of the implanting centre. The duration of machine perfusion will be dictated by logistics and local policy, but should not be less than 4 hours or more than 24 hours. The criteria for transplantation must be met within 4 hours. If cannulation proves impossible, the liver will be rejected as previously intended.

6.1 Stage 1: Liver Registration

Liver Registration will take place using the online registration system:
www.cancertrials.bham.ac.uk

Once a liver has been selected for entry into the clinical trial the following information will be recorded in the trial study database.

6.1.1 **Times to be recorded for DBD donors are as follows:**

- Cessation of donor circulation (cross clamp)
- Start of cold perfusion (should be the same unless technical problem)
- Liver removal and placement on ice
- Initiation of NMLP
- Cessation of NMLP (cold flush)

6.1.2 Times to be recorded for DCD donors are as follows:

- Liver removal and placement on ice
- Withdrawal of support
- Onset of functional warm ischaemia (SBP < 50 mmHg)
- Cessation of donor circulation
- Start of cold perfusion
- Initiation of NMLP
- Cessation of NMLP (cold flush)

6.1.3 Donor information

- Degree of steatosis (graded mild, moderate, severe) – surgeon's assessment
- Quality of in-situ cold-perfusion (graded poor, moderate, good)
- Perfusion solution used for aortic perfusion
- Perfusion solution used for PV perfusion
- PV perfusion performed in situ/on back-bench
- Perfusion solution used for organ transport
- Liver weight (if available)
- In addition to these pre-specified outcomes, additional biological samples will be taken -
    - 2 x 1ml perfusate samples (one arterial and 1 venous) will be taken for blood gas analysis before the liver is connected to the device (pre-perfusion) and every 30 minutes for the first 4 hours and every hour thereafter, during the perfusion.
    - Samples will be taken for WP2 at specified time-points as detailed in Appendix 6.
    - At the end of preservation, a sample of perfusate/storage solution will be taken for microbiological culture.

Thus, the aims of sample collection for WP2 are:

- Relate pathology to demographics, function and outcomes, and evaluate current criteria for viability
- Use of next generation mass-spectrometry to analyse relevant segments of the portal triad and of bile with genomics, proteomics and metabolomics techniques to identify candidate biomarkers and validate both new and existing markers predicting outcome of transplantation.
- To establish a simple assay for measurement in routine practice.
- To identify novel pathways of injury and repair in donor livers

6.2 Stage 2: Patient registration and transplant

An Eligibility Form must be completed before a patient is registered.
Patient registration will take place using the online registration system:
www.cancertrials.bham.ac.uk

6.2.1 Recipient information

Recipient demographics to be recorded will include the following:

- Age
- Sex
- Aetiology of liver disease
- Indication for transplant
- Model For End-Stage Liver Disease MELD score (based on International normalised ratio (INR), creatinine, bilirubin)
- UKELD
- BMI
- A baseline assessment of quality of life using the EQ-5D-5L will be performed at the time of initial consent to the study, which may be when the patient was first put on the liver transplant waiting list.

6.2.2 Normothermic Machine Liver Perfusion information

Time to be recorded –

- Initiation of NMLP
- Cessation of NMLP (cold flush)

Perfusion parameters (logged automatically by the device):

- Arterial and portal caval pressures (in mmHg)
- Arterial, portal and caval flow rates (in ml/min)
- pO_2, pCO_2 and pH
- Blood temperature (^oC)
- Glucose (mmol/L)
- Bile production (ml/h)

6.2.3 Operative information

- Start time
- Explantation time
- Anhepatic period
- Caval anastomosis start
- Portal reperfusion
- Arterial reperfusion
- Operation finish time

Using these totals - operating time and the anastomotic time [secondary warm ischaemia defined as time between removal of organ from the perfusion device to organ reperfusion] - can be calculated.

Operative parameters

- Presence of post-reperfusion syndrome (defined as a decrease in mean arterial pressure (MAP) of more than 30% from the pre-implantation baseline value for more than one minute during the first five minutes after reperfusion)
- Use of vasopressors prior to and after reperfusion
- Peak intraoperative lactate level

- Intraoperative transfusion of blood products (RBC, platelets, FFP, apropotin measured in units.
- The use of veno-venous bypass or porto-caval shunts
- Type of caval anastomosis (standard end-end, piggyback (end-side or side-side)

Histological evidence of ischaemia-reperfusion injury

- Graft biopsy (Liver Biopsy 4 as per Table 3) will be taken immediately prior to abdominal closure and examined for evidence of reperfusion injury.
- Histology report should be entered into database

These biopsies will be compared to the baseline biopsies prior to organ reperfusion and graded according to standard histological criteria. The trial histopathologists will assess all biopsies.

6.2.4 **Donor demographics**

Donor demographics to be recorded will include the following:

- Age
- Sex
- Race (White, Afro-Caribbean, other)
- Cause of death (CVA, hypoxia, trauma, other)
- Type of donor (DBD, DCD)
- Donor height
- Diabetes (UK-DRI)
- Smoking (UK-DRI)
- Donor risk index (DRI)
- Last and peak serum ALT / AST
- Last and peak serum sodium
- Last and peak GGT
- Bilirubin (used for UK-DRI)
- Albumin (for UK-DRI)
- Length of ITU (intensive therapy unit) stay
- BMI

6.2.5 **OrganOx *metra* ™ Information print-outs**

The following information will be recorded whilst the donor liver is attached to the OrganOx *metra* ™ device.

- Arterial and caval pressures (in mmHg)
- Arterial, portal vein and caval flow rates (in mmHg)
- pO_2, pCO_2 and pH
- Blood temperature (^{o}C)
- Glucose (mmol/L)
- Bile production (ml/h)

6.2.6 **Visit 1 – Transplant (Day 0) and Inpatient stay**

(Patient hospitalisation immediately post-operation through until day of discharge)

Recipient demographics

Recipient demographics to be recorded will include the following:

- Age
- Sex
- Aetiology of liver disease
- Indication for transplant
- MELD score (based on INR, creatinine, bilirubin)
- UKELD
- BMI
- A baseline assessment of quality of life using the EQ-5D-5L will be performed at the time of initial consent to the study, which may be when the patient was first put on the liver transplant waiting list.

Patients will be assessed daily by their clinical team and managed according to normal local protocols.

Outcome assessment:

The following biochemical outcomes will be recorded.

Daily serum samples for the first 7 days post-transplant, to include:

- Serum bilirubin (measured in μmol/L)
- Serum gamma-glutamyl transferase (GGT; measured in IU/L)
- Serum aspartate transaminase (AST; measured in IU/L)
- Serum alanine transaminase (ALT; measured in IU/L)
- Serum urea and creatinine levels
- International normalised ratio (INR)
- Daily serum lactate (measured in mmol/L)

The first measurements should be taken at 12 to 24 hours post-transplant. For subsequent measurements, in the event that more than one measurement is taken in a 24-hour period, the measurement taken closest to the specified time-point should be used.

For biological specimen collection see Appendix 6

Trial specific assessment:

PBMC (see Laboratory Manual)

Serum

Plasma

MN cells

Urine

Other outcomes to be recorded include:

- Length of ITU stay (days)
- Total length of hospital stay (days)
- Requirement for renal replacement therapy (RRT; haemodialysis (HD), haemodiafiltration (HDF), haemofiltration (HF))
- Graft and patient survival at day 7 post-transplant

- Primary non-function (classified as "irreversible graft dysfunction requiring emergency liver replacement during the first 10 days after liver transplantation, in the absence of technical or immunological causes.")

Safety outcomes:

- Recipient infection (defined as a positive microbiological culture result)
- Biopsy-proven acute rejection episodes
- Biliary complications (biliary strictures - anastomotic and non-anastomotic, bile duct leaks)
- Vascular complications (bleeding, hepatic artery stenosis, hepatic artery thrombosis, portal vein thrombosis, portal vein stenosis)
- Reoperation rate
- Concomitant medication used to treat any adverse event recorded as part of a serious adverse event
- Any other adverse event

Severity will be graded according to the Clavien-Dindo classification as described in Appendix 1.

Immunosuppression

Details of induction immunosuppression and maintenance immunosuppression (including doses) at day 7 post-transplant will be recorded.

Patient Resource Log to be given to patient at discharge or at next available clinic appointment.

6.3 Post-Transplant Visit Schedule

6.3.1 Visit 2 - Day 30 post-op (+/- 3 days)

This visit will, where possible, coincide with a routine outpatient appointment. If the recipient is an inpatient, assessment will be made in hospital where appropriate.

Outcome assessment:

The following biochemical outcomes will be recorded at day 30 post-transplant:

- Serum bilirubin (measured in µmol/l)
- Serum gamma-glutamyl transferase (GGT; measured in IU/L)
- Serum aspartate transaminase (AST; measured in IU/L)
- International normalised ratio (INR)

For biological specimen collection see Appendix 6

Trial specific assessment:

PBMC (see Laboratory Manual)

Serum (see Appendix 6)

Plasma (see Appendix 6)

MN cells (see Appendix 6)

Other outcomes to be recorded include:

- Graft and patient survival at day 30 post-transplant
- Requirement for renal replacement therapy (HD, HF, HDF) at any time
- Quality of life (measured by means of the EQ-5D-5L questionnaire).

Safety outcomes:

- Recipient infection (defined as a positive microbiological culture result)
- Biopsy-proven acute rejection episodes
- Biliary complications (biliary strictures - anastomotic and non-anastomotic, bile duct leaks)
- Vascular complications (bleeding, hepatic artery stenosis, hepatic artery thrombosis, portal vein thrombosis, portal vein stenosis)
- Reoperation rate
- Concomitant medication used to treat any adverse event recorded as part of a serious adverse event
- Any other adverse event

Severity will be graded according to the Clavien-Dindo classification as described in Appendix 1.

Immunosuppression

Details of maintenance immunosuppression (including doses) at day 7 and day 30 post-transplant will be recorded.

6.3.2 Visit 3 – 90 days post-op (+3 days)

This visit will, where possible, coincide with a routine outpatient appointment. If the recipient is an inpatient, assessment will be made in hospital where appropriate.

Outcome assessment

The following biochemical outcomes will be recorded at day 30 post-transplant:

- Serum bilirubin (measured in μmol/l)
- Serum gamma-glutamyl transferase (GGT; measured in IU/L)
- Serum aspartate transaminase (AST; measured in IU/L)
- International normalised ratio (INR)

For biological specimen collection see Appendix 6

Trial specific assessment:

PBMC (see Laboratory Manual)

Serum (see Appendix 6)

Plasma (see Appendix 6)

MN cells (see Appendix 6)

Other outcomes to be recorded include:

- Graft and patient survival at day 30 post-transplant
- Requirement for renal replacement therapy (HD, HF, HDF) at any time

Safety outcomes

- Recipient infection (defined as a positive microbiological culture result)
- Biopsy-proven acute rejection episodes
- Biliary complications (biliary strictures - anastomotic and non-anastomotic, bile duct leaks)
- Vascular complications (bleeding, hepatic artery stenosis, hepatic artery thrombosis, portal vein thrombosis, portal vein stenosis)
- Reoperation rate
- Concomitant medication used to treat any adverse event recorded as part of a serious adverse event
- Any other adverse event

Severity will be graded according to the Clavien-Dindo classification as described in Appendix 1.

Immunosuppression

Details of maintenance immunosuppression (including doses) at day 90 post-transplant will be recorded.

6.3.3 Visit 4 – 180 days post-op (+30 days)

This visit will, where possible, coincide with a routine outpatient appointment. If the recipient is an inpatient, assessment will be made in hospital where appropriate.

Outcome assessment:

The following biochemical outcomes will be recorded at month 6 post-transplant:

- Serum bilirubin (measured in µmol/l)
- Serum gamma-glutamyl transferase (GGT; measured in IU/L)
- Serum aspartate transaminase (AST; measured in IU/L)
- International normalised ratio (INR)

For biological specimen collection see Appendix 6

Trial specific assessment:

PBMC (see Laboratory Manual)

Serum (see Appendix 6)

Plasma (see Appendix 6)

MN cells (see Appendix 6)

Other outcomes to be recorded include:

- Graft and patient survival at month 6 post-transplant

- Requirement for renal replacement therapy (HD, HF, HDF) at any time
- Quality of life (measured by means of the EQ-5D-5L questionnaire)

All study participants will undergo magnetic resonance cholangiopancreatography (MRCP) with T2-weighted turbo-spin echo sequences at 6 months post-transplant unless contraindicated.

Contraindications to MRI include:

- Implanted pacemaker, defibrillator or metal heart valve
- Implanted pump device (such as an insulin or pain medication pump)
- Inner ear implant
- Aneurysm clip within the brain
- Intrauterine device (IUD)
- Metal in the eyes (at any time), or history of being a metal worker
- Current pregnancy
- Intractable claustrophobia

The trial radiologist will assess all MRCPs. Evidence of ischaemic cholangiopathy will be taken as the presence of extra-anastomotic biliary structuring in the absence of hepatic artery thrombosis.

Safety outcomes:

- Recipient infection (defined as a positive microbiological culture result)
- Biopsy-proven acute rejection episodes
- Biliary complications (biliary strictures - anastomotic and non-anastomotic, bile duct leaks)
- Vascular complications (bleeding, hepatic artery stenosis, hepatic artery thrombosis, portal vein thrombosis, portal vein stenosis)
- Reoperation rate
- Concomitant medication used to treat any adverse event recorded as part of a serious adverse event
- Any other adverse event

Severity will be graded according to the Clavien-Dindo classification as described in Appendix 1.

Immunosuppression

Details of maintenance immunosuppression (including doses) at 6 months post-transplant will be recorded.

6.4 Extended follow up – 12 month post - transplant (+/- 30 days) and 24 month post – transplant (+/- 30 days)

Whilst the end-point for trial participation will be 6 months, we also intend to collect graft and patient survival data, adverse event information and serum biochemistry values from consenting participants at 12 and 24 months post-transplant. These data will be measured as

part of routine clinical care, not requiring additional visits or interventions on the part of the trial participant.

The biochemical parameters to be recorded are:

- Serum bilirubin (measured in μmol/l)
- Serum gamma-glutamyl transferase (GGT; measured in IU/L)
- Serum aspartate transaminase (AST; measured in IU/L)
- International normalised ratio (INR)

Safety outcomes:

- Concomitant medication used to treat any adverse event recorded as part of a serious adverse event
- Any other adverse event

Severity will be graded according to the Clavien-Dindo classification as described in Appendix 1.

6.5 Assessments

6.5.1 Blood chemistry and haematology as part of standard of care

	**Blood test**
**Biochemistry**	Alkaline Phosphatase (ALP)
	Alanine Transaminase (ALT)
	Aspartate Transaminase (AST)
	Albumin
	Bilirubin
	Gamma Glutamyl Transferase (GGT)
	Sodium
	Potassium
	Urea
	Creatinine
	Calcium
	Total Protein
**Haematology**	Haemoglobin
	Platelets
	Red Blood Cells
	White Blood Cells
	Haematocrit
	Mean Cell Volume

	Mean Cell Haemoglobin
	Neutrophils
	Lymphocytes
	Monocytes
	Eosinophils
	Basophils
	INR

6.6 Evaluation of potential biomarkers of graft viability (Work Package 2)

Serum, plasma and immune cell isolation

3 additional blood samples will be taken at the same time as the patients standard blood tests. These will be processed to isolate the patient's serum, plasma and mononuclear cells. These samples will be used along with the perfusate and tissue samples as part of WP2 to identify potential biomarkers of graft viability.

6.6.1 Histological analysis of tissue samples obtained during NMLP and following transplantation.

Methodology: Biopsy collection and histological evaluation: Liver Menghini biopsies will be obtained at the following four time points

1. Pre-perfusion on back bench
2. After 4 hours of perfusion (when decision is made regarding suitability for transplantation)
    - This is to standardise the time-point for the perfusion biopsy
3. Post-perfusion/pre-implantation
    - This will be performed at the end of the perfusion only if the perfusion lasts longer than 6 hours.
    - Some perfusions will likely last greater than 6 hours and it is therefore important to get a picture of the histology at the end of the perfusion
4. Post-implantation following reperfusion and prior to abdominal closure (if liver meets criteria for transplantation).

Where possible (duct length permitting), donor bile duct biopsies will be obtained pre-perfusion on the back bench and prior to formation of the bile duct anastomosis. Fresh frozen and formalin fixed/paraffin embedded tissue samples will be processed. A small piece of tissue will be fixed in glutaraldehyde for electron microscopy.

The tissue samples collected will be analysed using a range of histological techniques including conventional light microscopy, electron microscopy and immunohistochemistry to investigate the presence and severity of various histological features that may be important in the assessment of graft viability and preservation-reperfusion injury (PRI) including fatty change, neutrophil infiltration, glycogen content, hepatocyte apoptosis/necrosis and mitochondrial injury. Where applicable, quantitative assessment using digital algorithms on whole slide images will also be carried out. Any changes seen in livers transplanted following NMLP will be compared with those present in livers that are not viable after NMLP and with those seen in biopsies taken from contemporaneous "traditional" liver transplants at UHB.

Findings will be correlated with outcomes including early post-operative AST as a marker of hepatocellular injury from PRI, primary non-function or delayed graft function and the development of biliary complications.

6.6.2 Biochemical analysis and other studies of perfusate samples generated during NMLP and following transplantation.

Biochemical analysis of perfusion fluid will be carried out to identify and validate potential biomarkers of graft viability. These data will be obtained from the analysis of perfusates collected throughout the perfusion period and from recipient blood samples following transplantation as defined in Appendix 6. Samples will be stored in the WP2 bioresource and used for approved related studies.

6.7 Treatment Compliance

Patients will receive standard care as per local guidelines following transplantation. Treatment compliance will be monitored through standard care.

6.8 Supportive Treatment

There are no additional protocol defined procedures relating to the liver transplant operation and the local standard practice for both pre-operative and post-operative care will be followed. This care and supportive treatment will be fully documented in the patient records and subsequently reported in the individual patient case report form.

6.9 Concomitant Medication

Recipient management including the implantation procedure, post-operative care, immunosuppression and other medications, and post-transplant monitoring will follow local procedure.

Only the following concomitant medication information will be recorded throughout the clinical trial.

Immunosuppression - Details of maintenance immunosuppression (including doses) post-transplant will be recorded on the case report form / eRDC trial system. No other concomitant medication will be recorded except during this clinical trial as part of the case report form except;

1. Any medication that was prescribed / used to treat any adverse event recorded as part of a serious adverse event – irrespective of adverse event causality. This information will be recorded on both the case report form and as part of the serious adverse event form.

6.10 Health economic analysis

An economic analysis will be performed by a Health Economist, with the objective of estimating average costs and effectiveness of viability testing using NMLP. This will inform a cost-effectiveness analysis using a health service perspective and incremental cost effectiveness ratios (ICER's) will be reported. Quality adjusted survival will be obtained by administration of the EuroQol EQ-5D-5L questionnaire (Appendix 2). Quality of life data will be collected at baseline (pre-transplant, at time of consent) and at each study follow-up visit following liver transplantation (day 7, day 30 and month 6). Costs will be estimated based upon measured resource use and national unit costs. Resources will include machine and disposables costs, immunosuppression and other drugs, inpatient hospital stays (including intensive care days), radiological investigations, biopsies and other procedures, outpatient visit and visits to the family doctor. Resource use will be identified from case report forms,

hospital episode statistics/insurer claims and from patient self-reporting using a simple log/questionnaire (to assess out-of hospital resource use). These questionnaires will be kept by the patient during the study and collected at the final study visit.

Resource use will be transferred to an electronic Case Report Form (eCRF), and the original document kept at the participating centre as source material

6.11 Patient Retention and Withdrawal

All registered patients (transplant recipients) completing the 6-month follow-up assessment will be regarded as having completed the primary study. All patients will be encouraged to complete study follow-up, and all reasonable efforts will be made to ensure completeness of follow-up. Measures include ensuring that assessments are made, where possible, at routine hospital visits rather than additional appointments and that patients' do not incur extra financial costs (e.g. travelling costs) as a result of study participation.

It is understood that study participants may withdraw consent for study participation at any time irrespective of their reasons. The investigators may also withdraw a recipient from the study in order to protect their safety and/or if they are unwilling or unable to comply with the required study procedures. We will keep all data accrued to the point of withdrawal unless the participant requests otherwise, as is stipulated in the trial consent form.

Possible reasons for investigator-led withdrawal of a participant from the trial include:

- Ineligibility either arising during the trial or retrospectively having been overlooked at screening
- Significant protocol deviation
- Significant non-compliance with trial requirements
- An adverse event which leads to inability to comply with trial procedures
- Disease progression which results in an inability to comply with trial procedures
- Withdrawal of consent
- Loss to follow-up

In the event a patient withdrawing from the trial, the reason for withdrawal must be documented on the eCRF. Such patients will be asked whether they consent to data accrued before the date of withdrawal being included in the trial analysis.

7 INTERVENTION DETAILS

7.1 Device Description- OrganOx *metra* ™

The OrganOx *metra* ™ is a normothermic preservation device for use in human liver transplantation. It perfuses the donor liver with blood, oxygen and nutrients, as well as a number of medications, at normal body temperature to mimic ideal physiological conditions and preserve the organ for up to 24 hours. The device provides information as to the haemodynamic, synthetic and metabolic function of the liver whilst being perfused which we aim to show can assist the clinician in assessing the organ's suitability for transplantation.

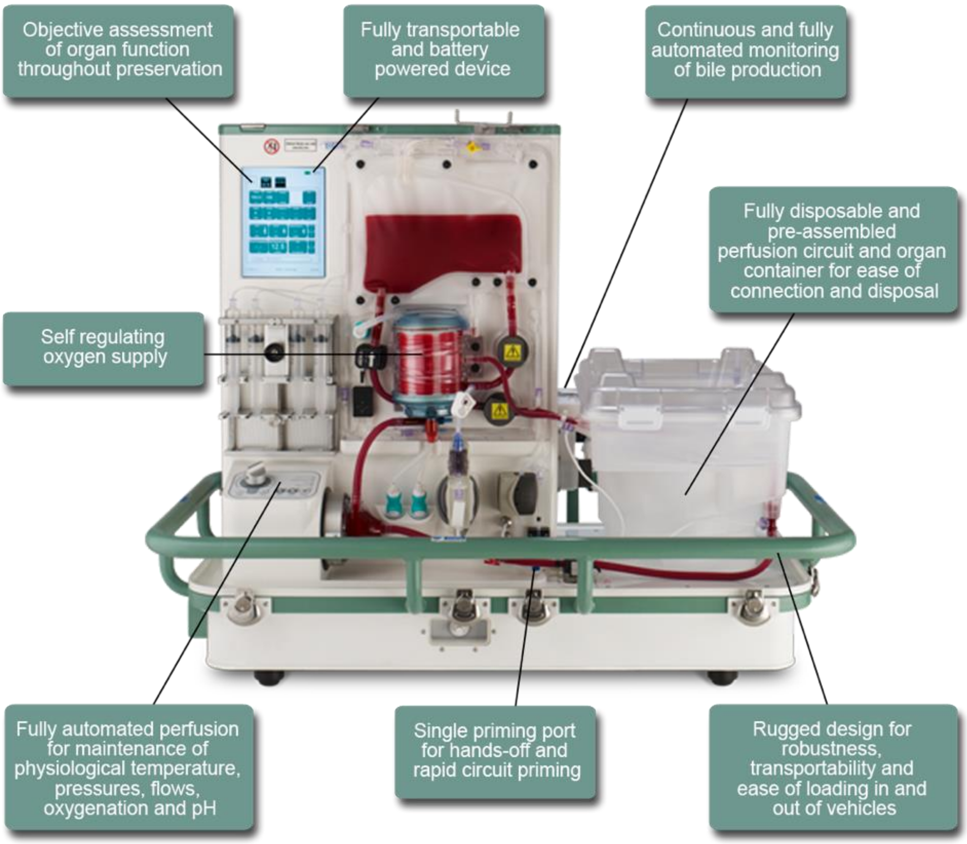

Figure 4: OrganOx *metra* ™ Machine

7.2 Manufacturing Organisation

OrganOx Limited is a late-stage medical device development company that was founded in April 2008 as a spin-out from the University of Oxford.

7.3 The OrganOx *metra* ™ Base Unit

The OrganOx *metra* ™ normothermic perfusion device incorporates a centrifugal pump, an oxygenator, oxygen concentrator, heat exchanger, reservoir, flow probes, pressure sensors, infusions and blood gas analyser together with tubing and connector components. The device is comprised of three main components:

- a reusable base unit which contains software and hardware
- a disposable plastic circuit
- a set of perfusion solutions suitable for 24 hours perfusion

7.3.1 **Disposable set**

The disposable set used with the core base unit of the OrganOx *metra* ™contains all the disposables used with each organ recovery on the *metra* and comprises:

- A disposable tubing set, including a blood reservoir, perfusion lines, a blood oxygenator and centrifugal pump-head together with flow and pressure sensors.
- An organ storage bowl which is pre-connected to the tubing set to contain the organ while on the device.

- Cannulae for the coeliac artery, portal vein and inferior vena cava with easy connection attachment to the perfusion circuit.
- A cannula and connection point for bile collection
- Blood gas sensors for monitoring pO_2, pCO_2 and pH by means of on-line blood gas analysis.

7.3.2 Perfusion solutions

For VITTAL, all the additives necessary to perfuse and maintain the organ during the storage process, will be sourced from UHBFT. These solutions include bolus injections (given at the start of perfusion) and the maintenance infusions (given throughout perfusion).

The primary perfusion fluid for the liver comprises packed red blood cells, supplemented by colloid solution to normalise the haematocrit and osmolarity.

Before connection of the liver the blood-based perfusate is supplemented with:

- Gentamicin (antibiotic). Antibiotic use may be tailored according to specific sensitivities identified in donor cultures or if concerns exist about potential contaminants.
- Heparin (anticoagulant) to prevent thrombosis in the circuit. In clinical use, a half-life of ~90 minutes is assumed; on this basis heparin is also given as a maintenance infusion.
- Sodium bicarbonate (buffer) for adjusting the pH of the perfusate before the liver is placed on the device.
- Calcium gluconate to correct the binding of citrate to calcium. During the perfusion the following are infused at a constant rate:
- Parenteral nutrition solution - a source of amino acids and glucose for liver maintenance. Insulin to control the perfusate glucose level
- Heparin to maintain anticoagulation.
- A 2% solution of sodium taurocholate in isotonic saline to compensate for loss of bile salts.
- Prostacyclin to optimise micro-perfusion.

The primary fluid for perfusing the organ will be packed red cells supplied by the blood bank at UHB and supplemented by a commercially-available colloid solution to normalise the haematocrit and osmolarity. All solutions required will be attached to the circuit during set-up and before the liver is attached.

7.4 Device safety

In designing the OrganOx *metra*, OrganOx Ltd has made every attempt to maintain the current practices of organ retrieval and transplant teams, in order to minimise the risk of complications or errors that would prevent a successful retrieval. From a regulatory standpoint, it is important to note that the *metra* is an organ preservation system and its use does not involve direct connection to either the donor or recipient at any time. The device has been designed according to ISO 13485, the standard that stipulates the requirements for a comprehensive management system for the design and manufacture of medical devices. In addition ISO 14971 specifies a process for a manufacturer to identify the hazards associated with medical devices to estimate and evaluate the associated risks, to control these risks,

and to monitor the effectiveness of the controls. As part of the development of the device an extensive risk analysis has been undertaken and the risks identified and minimised in accordance with this standard. As a result, any remaining risk can only be investigated by a clinical transplant study. The OrganOx *metra* ™ perfusion system is based on the principle that all the perfusion solutions, additives and packed red cells must be removed from the organ prior to transplant. Therefore following the completion of the perfusion, the perfusion solution is flushed out of the organ with HTK solution. OrganOx Ltd has deliberately designed the operation of the device such that it will require minimal changes to current transplant clinical practice. OrganOx *metra* ™ is CE marked.

7.5 Device labelling

All components of the OrganOx *metra* ™ system (reusable base unit and disposable set) will be labelled by OrganOx Ltd as “Exclusively for Clinical Investigation”. Labelling will also include the Sponsor name, contact details and a unique trial identifier.

7.6 Device accountability

Device accountability will be undertaken at the each local site throughout the study for the reusable unit(s) and disposable sets (sterilisation/assembly batch number and disposable set number). The manufacturer and lot number for each perfusion solution will also be recorded on the case report forms (CRFs). The site will maintain a log of usage of both the retained unit, disposable set and perfusion solutions used throughout the study recording the lot number used against each subject (on the CRF).

At the end of each procedure the OrganOx *metra* ™ and any unused disposable and perfusion solutions will be removed from the donor hospital and returned to the investigator centre. Details of total numbers of disposable sets taken on-site and off-site will also be recorded.

7.7 Device maintenance

Device cleaning and routine maintenance will be the responsibility of the local investigator storing the device. Full details for cleaning and routine maintenance required will be provided in the relevant section of the Instructions for Use (IFU).

7.8 Device logistics

7.8.1 Logistical considerations

For the purposes of this trial the only logistical consideration is that the device requires the manual assessment and input of perfusate glucose every 4 hours during perfusion.

7.8.2 Logistical arrangements

The OrganOx *metra* ™ device will be stored and maintained at UHBFT. When an eligible rejected donor organ is offered and confirmed by two UHB consultant surgeons, the local recipient co-ordinator/surgeon will check to see if there is a suitable consented recipient to take part in the study. If this is the case, then the coordinator will check whether the OrganOx *metra* ™ device and support team are available (it will not be possible to support multiple retrievals simultaneously as there will only be a single device). If the device and support team are available, then the named recipient will be contacted, informed consent confirmed verbally over the telephone, and checks made that the recipient still fulfils all

inclusion/exclusion criteria. The recipient co-ordinator/surgeon will then contact the lead retrieval surgeon to inform him/her that the liver is to be used in VITTAL. The research fellow will arrange for 3 units of recipient-type red blood cells to be cross-matched at UHB for use in the OrganOx *metra*™ device as previously described. Following retrieval, the liver will be transported to UHB and placed on the OrganOx *metra*™.

8 ADVERSE EVENT REPORTING

The collection and reporting of Adverse Events (AEs) will be in accordance with the Research Governance Framework for Health and Social Care and the requirements of the National Research Ethics Service (NRES). Definitions of different types of AE are listed in Appendix 7. The Investigator should assess the seriousness and causality (relatedness) of all AEs experienced by the patient (this should be documented in the source data) with reference to the protocol.

8.1 Reporting Requirements

8.1.1 **Adverse Events, Device Deficiencies and Adverse Device Effects**

All medical occurrences which meet the definition of an AE, Device Deficiencies and Adverse Device Effect (ADE), (see Appendix 7 for definitions) should be recorded and reported. Please note this includes abnormal laboratory findings which are reported as clinically significant.

All AEs, Device Deficiencies and ADEs will be reported using the applicable CRF/eRDC form. AEs will be reported in accordance with Clavien-Dindo classification of surgical complications (see Appendix 1).

Anticipated AEs are listed below; however this is not an exhaustive list.

General AEs

- Infection (chest, urine, blood, bile, wound, abdominal)
- Fluid collection (abdominal, pleural)
- Rejection
- Renal dysfunction
- Hepatic dysfunction
- Cardiac failure
- Respiratory failure

AEs related to the disease / condition / surgery

- Early graft dysfunction
- Admission for suspected rejection
- Occurrence and treatment of abdominal or wound infection
- Respiratory failure requiring mechanical ventilation
- Hospitalisation for pre-existing condition that has not deteriorated.
- Clinically significant abnormal laboratory finding or other abnormal assessments that is associated with the condition being studied (unless judged by the investigator as more severe than expected for the patient’s condition).

The investigator will exercise his/her medical judgment in deciding whether an abnormal laboratory finding or other abnormal assessment is clinically significant. However, if in the

opinion of the investigator, the frequency or severity of the event is greater than would be expected then it must be reported.

Device deficiencies that did not lead to an adverse event, but could have led to a medical occurrence if suitable action had not been taken, or intervention had not been made or if circumstances had been less fortunate will also be recorded and reported.

8.1.2 Known liver transplant post-operation complications

Complications			Incidence (%)
Acute Rejection	Requiring biopsy or medication		25%
Primary Non Function	Requiring re-transplantation		4%
Initial poor function			16%
Kidney dysfunction	Requiring alteration of medication up to dialysis		35%
Infectious complications	Including wound, urinary tract, blood stream infections, pneumonia and cholangitis requiring antibiotic treatment		
Bleeding Complications	Surgical site bleeding requiring reoperation or transfusion		10%
Biliary Complications	Biliary leakage requiring drainage or endoscopy		10%
	Anastomotic biliary strictures requiring intervention		15%
	Non-anastomotic biliary strictures requiring intervention		10%
Thrombotic and Ischemic Complications	Other venous thrombotic events (portal vein thrombosis or stenosis, deep venous thrombosis, pulmonary embolism)		3%
	Hepatic artery thrombosis requiring intervention or re-transplantation		6%
	Cardiovascular events	Requiring medication or electroversion	6%
		Infarction	2%
		Arrhythmia	3%
		New congestive heart failure	2%
	Cerebrovascular events		3%
		Stroke	2%
		Hemorrhage	0,3-0,6%
Central nervous system	Including delirium requiring medication		20%
NODAT	Requiring insulin		7%
Death	3 months mortality		7%

Table 4 List of known complications

8.1.3 Serious Adverse Events and Serious Adverse Device Effect

Investigators should report AEs and ADEs that meet the definition of a Serious Adverse Event (SAE) and Serious Adverse Device Effect (SADE) respectively (see Appendix 7 for definitions) and are not excluded from the reporting process as described in section 8.1.5. These need to be recorded on a SAE Form in addition to the Adverse Event CRF/eRDC Form. All SAEs need to be submitted to the study sponsor via the VITTAL Trial Office within 24 hours of site staff becoming aware of the event.

8.1.4 **Events that do not require expedited reporting**

All AEs that occur from the patient entry into the trial (defined as day of transplant operation) need to be recorded both in the patient medical records and Case Report Form (CRF/eRDC system).

8.1.5 **Events that do not require reporting on a Serious Adverse Event Form**

All AEs will be reported but the following are routine after liver transplantation and will not be Considered SAEs or require expedited reporting:

- Initial admission to Intensive Care following liver transplant
- Elevation of AST and/or ALT <2000 iu/ml within 48 hours of liver transplant
- Transfusion up to and including 5 units of packed red cells
- Transfusion of up to and including 8 units of fresh frozen plasma
- Transfusion of up to and including 2 adult doses of platelets

An overnight stay in hospital that is due to transportation, organisational or accommodation problems, and without medical background, does not need to be documented as a SAE.

In addition to the above, medical and scientific judgement should be exercised in deciding whether expedited reporting is appropriate in other situations, such as important medical events that may not be immediately life threatening or result in death or hospitalisation but may jeopardise the patient or may require intervention to prevent one of the outcomes listed in the definitions detailed in Appendix 7.

Any death occurring during the protocol defined follow up period (within 90 days), whether considered device-related or not, must be reported as an SAE within 24 hours of the local investigator becoming aware of the event. If a death occurs in a patient receiving a transplant the cause of death will be investigated and reviewed by the Trial Management Group (TMG) and clinical team caring for the patient. Entry of patients in to the study would be temporarily suspended until these investigations are complete.

Planned hospitalisation for a pre-existing condition, or a procedure required by the trial protocol, without serious deterioration in health, is not considered a serious adverse event.

In addition to notifying the appropriate regulatory agencies all SAE events that occur during this study and are related to the OrganOx *metra* ™ device (Serious Adverse Device Effect (SADE) and USADEs) will be reported to OrganOx Ltd Pharmacovigilance department (for information only) by the CRCTU VITTAL study clinical trial team, within 48 hours of the team becoming aware of the event.

The form will be completed and signed by the investigator and faxed to the trial office immediately. In signing of the SAE form the investigator is confirming the causality assessment.

All SAEs that are at least possibly related to the OrganOx *metra* ™ device – SADE) - still present at the end of the study will be followed at least until the final outcome is determined. Even if it implies that the follow-up continues after the patients leave the trial and when appropriate until the end of the planned period of follow-up

Within 30 days following the anniversary of the authorization date for the trial an Annual Safety Report will be sent by the Chief Investigator to the MHRA and the Main Research Ethics Committee. A copy of the report will also be sent to the sponsor and OrganOx Ltd.

8.1.6 Provision of follow-up information

Patients should be followed up until resolution or stabilisation of the event. Follow-up information should be provided on a new SAE Form (refer to the SAE Form Completion Guidelines for further information).

8.1.7 Trials Office

On receipt of an SAE Form seriousness and causality will be determined independently by a Clinical Coordinator. An SAE judged by the Investigator or Clinical Coordinator to have a reasonable causal relationship with the OrganOx *metra* ™ medical device will be regarded as a related SAE. The Clinical Coordinator will also assess all related SAEs for expectedness. If the event is unexpected (i.e. is not defined in the protocol as an expected event) it will be classified as an unexpected and related SAE.

8.2 Reporting to the Competent Authority and main Research Ethics Committee

8.2.1 Adverse Events and Adverse Device Effects

Details of all AEs will be reported to the MHRA on request.

8.2.2 Serious Adverse Device Effect

The Trials Office will report details of all SADEs (including USADEs) to the MHRA and main REC annually from the date of the Clinical Trial Authorisation, in the form of an Annual Safety Report.

8.2.3 Serious Adverse Events

The VITTAL Trials Office will report all events categorised as Unexpected and Related SAEs to the main Research Ethics Committee (REC) within 15 days. All SAEs regardless of causality or relatedness, excluding those listed in section 8.1.5, will be reported to the MHRA within 7 days of receipt from Site.

8.2.4 Unanticipated Serious Adverse Device Effects

The Trials Office will report a minimal data set of all USADEs to the Medicines and Healthcare products Regulatory Agency (MHRA) and main Research Ethics Committee (REC) within 7 days. Detailed follow-up information will be provided as appropriate.

All other events categorised as USADEs will be reported within 7 days.

8.2.5 Other safety issues identified during the course of the trial

The MHRA and main REC will be notified immediately if a significant safety issue is identified during the course of the trial.

8.2.6 Investigators

Details of all Unexpected and Related SAEs, USADEs and any other safety issue which arises during the course of the trial will be reported to Principal Investigators. A copy of any such correspondence should be filed in the ISF.

8.2.7 Reporting period

The reporting period for AEs will commence at Day 1 and will continue until 90 days post liver transplantation. SAEs will be reported until day 90 of the trial.

8.2.8 Data Monitoring Committee

The independent Data Monitoring Committee (DMC) will review all SAEs.

8.3 Reporting Procedure

All SAEs and (SADEs) will be reported to the VITTAL Trial Office within 24 hours of the investigator becoming aware of the event. SAEs will be documented on the SAE Form, which will be faxed within 24 hours to VITTAL Trial Office on the following number;

SAE FAX NO:
0121 371 8028 (Primary fax number)
0121 414 2230 (Secondary fax number)

The SAE Form will be completed and signed by the investigator and faxed to the trial office immediately. In signing of the SAE form the investigator is confirming the causality assessment.

In addition, ADEs and SADEs, as defined in Appendix 7, that occur during this study and are related to the OrganOx *metra* ™ device will be reported to OrganOx Ltd Pharmacovigilance department (for information only) by the VITTAL Trial Office within 48 hours of the team becoming aware of the event.
All ADEs that are at least possibly related to the OrganOx *metra* ™ device which are still present at the end of the study will be followed at least until the final outcome is determined. Follow-up of these ADEs will continue after the patients leave the trial and when appropriate until the end of the planned period of follow-up.

Within 30 days following the anniversary of the authorisation date for the trial an Annual Safety Report will be sent by the Chief Investigator to the MHRA and the Main Research Ethics Committee. A copy of the report will also be sent to the sponsor and OrganOx Ltd.

8.3.1 Provision of follow-up information

Patients should be followed up until resolution or stabilisation of the event. Follow-up information should be provided on a new SAE Form (refer to the SAE Form Completion Guidelines for further information).

8.3.2 Trials Office

On receipt of an SAE Form seriousness and causality will be determined independently by a Clinical Coordinator. An SAE judged by the Investigator or Clinical Coordinator to have a reasonable causal relationship with the OrganOx *metra* ™ medical device will be regarded

as a related SAE. The Clinical Coordinator will also assess all related SAEs for expectedness. If the event is unexpected (i.e. is not defined in the protocol as an expected event) it will be classified as an unexpected and related SAE.

9 DATA HANDLING AND RECORD KEEPING

9.1 Data Collection

The Case Report Form (CRF) will comprise the following forms:

Form	Summary of data recorded	Schedule for submission
Eligibility Checklist	Confirmation of eligibility and satisfactory staging investigations where necessary	Faxed at point of registration
Liver Registration	Donor details, donor liver details,	At time of liver registration
Perfusion Form	Perfusion details	After liver registration, before patient registration
Donor History Form	Donor history details	As soon as possible after liver registration
Patient Registration	Recipient details	At time of patient registration
Visit Forms (Transplant Form, Biochemistry Form, Morbidity Form, MRCP Form)	Patient data, assessment data as per visit schedule	As soon as possible after visit date
Follow Up Forms	Patient data, assessment data as per visit schedule	As soon as possible after visit date
Death Form	Date and cause of death	Immediately upon notification of patient's death
Deviation Form	Completed in the event of a deviation from the protocol	Immediately upon discovering deviation
Withdrawal Form	Used to notify the Trials Office of patient withdrawal from the trial	Immediately upon patient withdrawal

Table 5 Case Report Forms (CRFs)

Ad hoc forms

Serious Adverse Event Form

Death Report Form

The CRF must be completed, signed/dated and returned to the Trials Office by the Investigator or an authorised member of the site research team (as delegated on the Site Signature and Delegation Log) within the timeframe listed above.

Entries on the CRF should be made in ballpoint pen, in blue or black ink, and must be legible. Any errors should be crossed out with a single stroke, the correction inserted and the

change initialled and dated. If it is not obvious why a change has been made, an explanation should be written next to the change.

Data reported on each form should be consistent with the source data or the discrepancies should be explained. If information is not known, this must be clearly indicated on the form. All missing and ambiguous data will be queried. All sections are to be completed before returning.

In all cases it remains the responsibility of the Investigator to ensure that the CRF has been completed correctly and that the data are accurate.

Trial forms may be amended by the Trials Office, as appropriate, throughout the duration of the trial. Whilst this will not constitute a protocol amendment, new versions of the form must be implemented by participating sites immediately on receipt.

10 ARCHIVING

It is the responsibility of the Principal Investigator to ensure all essential trial documentation and source records (e.g. signed Informed Consent Forms, Investigator Site Files, Pharmacy Files, patients' hospital notes, copies of CRFs etc) at their site are securely retained for at least 15 years. Do not destroy any documents without prior approval from the CRCTU Document Storage Manager.

11 QUALITY MANAGEMENT

The VITTAL clinical trial is being conducted under the auspices of the Cancer Research UK Clinical Trials Unit (CRCTU) according to the current guidelines for Good Clinical Practice (GCP). Participating sites will be monitored by CRCTU staff to confirm compliance with the protocol and the protection of patients' rights as detailed in the Declaration of Helsinki: October 1996 (Appendix 8).

11.1 Site Set-up and Initiation

All sites will be required to sign a Clinical Study Site Agreement prior to participation. In addition all participating Investigators will be asked to sign the necessary agreements including Investigator Registration Form, Site Signature and Delegation Log and supply a current CV to the Trials Office. All members of the site research team will also be required to sign the Site Signature and Delegation Log, which should be returned to the Trials Office. Prior to commencing recruitment all sites will undergo a process of initiation. Key members of the site research team will be required to attend either a meeting or a teleconference covering aspects of the trial design, protocol procedures, Adverse Event reporting, collection and reporting of data and record keeping. Sites will be provided with an Investigator Site File containing essential documentation, instructions, and other documentation required for the conduct of the trial. The Trials Office must be informed immediately of any change in the site research team.

11.2 On-site Monitoring

Monitoring will be carried out as required following a risk assessment and as documented in the VITTAL Quality Management Plan. Additional on-site monitoring visits may be triggered for example by poor CRF return, poor data quality, low SAE reporting rates, excessive number of patient withdrawals or deviations. If a monitoring visit is required the Trials Office will contact the site to arrange a date for the proposed visit and will provide the site with

written confirmation. Investigators will allow the VITTAL trial staff access to source documents as requested.

11.3 Central Monitoring

Where a patient has given explicit consent sites are requested to send copies of signed Informed Consent Forms to the VITTAL Trial Office for in-house review.

The VITTAL trial staff will be in regular contact with the site research team to check on progress and address any queries that they may have. Trials staff will check incoming Case Report Forms for compliance with the protocol, data consistency, missing data and timing. Sites will be sent Data Clarification Forms requesting missing data or clarification of inconsistencies or discrepancies.

Sites may be suspended from further recruitment in the event of serious and persistent non-compliance with the protocol and/or GCP, and/or poor recruitment. Any major problems identified during monitoring may be reported to the Trial Management Group and the relevant regulatory bodies. This includes reporting serious breaches of GCP and/or the trial protocol to the main Research Ethics Committee (REC) and the Medicines for Healthcare products Regulatory Agency (MHRA).

11.4 Audit and Inspection

The Investigator will permit trial-related monitoring, audits, ethical review, and regulatory inspection(s) at their site, providing direct access to source data/documents.

Sites are also requested to notify the Trials Office of any MHRA inspections.

11.5 Notification of Serious Breaches

The Sponsor of the trial is responsible for notifying the licensing authority in writing of any serious breach of:

1. The conditions and principles of GCP in connection with that trial or;
2. The protocol relating to that trial, within 7 days of becoming aware of that breach

For the purposes of this regulation, a “serious breach” is a breach which is likely to effect to a significant degree:

3. The safety or physical or mental integrity of the subjects of the trial; or
4. The scientific value of the trial

Sites are therefore requested to notify the Trials Office of a suspected trial-related serious breach of GCP and/or the trial protocol. Where the Trials Office is investigating whether or not a serious breach has occurred sites are also requested to cooperate with the Trials Office in providing sufficient information to report the breach to the MHRA where required and in undertaking any corrective and/or preventive action.

12 END OF TRIAL DEFINITION

Data will be collected from participants for 24 months post-transplant. However, the defined Last Patient: Last Visit (LPLV) (study endpoint) is expected to be 6 months (180 days) after the last patient (patient 22) has received a liver transplant operation at which point they will

undergo MRCP and outpatient follow-up. This will allow sufficient time for the completion of protocol procedures, data collection and data input. Therefore the end of trial definition will be when all patients have either reached the 6 month follow-up time point or have been withdrawn from the clinical trial prior to this data collection point for other reasons i.e. Patient death.

The Trials Office will notify the main REC and MHRA that the trial has ended and a summary of the clinical trial report will be provided within 12 months of the end of trial. Long term follow up visits are scheduled for 12 months and 24 month post-transplant. The data collected will be primarily focusing on patient survival therefore supplementary reports may be provided to the MHRA at a later date.

Once all data has been processed, the database will be locked and the trial will end. It is anticipated that the ethics approval will be maintained for approximately 24 months post the LPLV to enable all of the samples to be processed. Any remaining samples after this time will either be transferred to an HTA licenced tissue bank or 'transferred' and stored under another ethical approved project subject to individual patients and donor consent.

13 STATISTICAL CONSIDERATIONS

This is a non-randomised, prospective, single arm study designed with two linked components with co-primary aims of:

(A) establish the feasibility of NMLP as a means to increase the number of transplantable livers using a 2-stage design

(B) Achievement of successful transplantation using rejected liver grafts assessed using a 3-stage design

Part (A) requires up to 53 marginal livers to be entered into the trial, whereas part (B) requires 22 transplantations of viable livers to be performed. Following transplantation of the 22nd liver, no further livers will be considered for transplantation however livers can still be perfused and entered into work package 2 and of course details will be recorded of how the liver performed on the machine and whether the liver would have been considered viable.

13.1 Determination of Sample Size

For (A), it is anticipated that NMLP will achieve a desirable organ recovery rate of at least 50%, with an undesirable rate of 30% or less as it will not be economically feasible. The significance level (α) is set at 0.05, corresponding to the probability of incorrectly rejecting H_0 given it is true (Type I error), and the power is set at 0.90 (Type II error rate, $\beta = 0.10$), corresponding to the probability of correctly deciding the NMLP treatment is successful given the true response rate is greater than 50%.

Using a **Simon's two-stage design** [16]

Stage 1A of accrual: 24 marginal grafts will be perfused and assessed in the first stage. The procedure is infeasible if there are fewer than 8 recovered livers. Otherwise, proceed to Stage 2A.

Stage 2A of accrual: Up to additional 29 marginal grafts will be perfused. We would accept the procedure as feasible if there are at least 22 recovered livers out of **53 perfused livers**.

For (B), for viable livers following NMLP, a desirable 90-day patient survival rate is at least 88%, with an undesirable rate of 73% (15% lower). The mean 90-day patient survival rate for ‘standard’ liver transplants is 93% (ref Annual Report on Liver Transplantation, NHS Blood and Transplant, 2014)[18].

An **optimal three-stage design[17]** will be used to test the null hypothesis that the mean 90-day patient survival rate will be less than 73% ($P \leq 0.73$), versus an alternative hypothesis - that the 90-day patient survival rate will be at least 88% ($P \geq 0.88$).

The significance level is set at 0.20 (target $\alpha=0.2$), giving a 0.2 probability to conclude that a single transplantation is viable when it truly is not viable.

The power is set at 80% (target $\beta=0.2$), giving a 0.2 probability to conclude that a single transplantation is not viable when it truly is viable.

Stage 1B: Following transplantation in 3 patients, the trial will stop early (concluding $P \leq 0.73$) if there are fewer than 2 patients achieving 90-day survival. Otherwise an additional 8 transplantations will be performed.

Stage 2B occurs after transplantation in 11 patients (combined first and second stages) the trial will stop early (concluding $P \leq 0.73$) if there are seven or fewer successes, otherwise an additional 11 transplantations will be performed.

At Stage 3B, after transplantation in **22 patients** in all three stages, the trial will conclude successful achievement of successful transplantation of previously rejected donor liver following NMLP if there are at least 18 patients achieving 90-day survival.

The trial schema is provided in Figure 1.

13.2 Definition of Objectives and Outcome Measures

See Section 2.2.

13.3 Analysis of Outcome Measures

13.3.1 Primary Analysis

To assess (A), the feasibility of NMLP, we evaluate the rescue rate, which will be calculated as the number of perfused marginal grafts meeting the criteria for viability (numerator) divided by the total number of perfused marginal grafts (denominator).

$$Rescue\ Rate = \frac{Number\ of\ viable\ perfused\ marginal\ grafts}{Total\ number\ of\ perfused\ marginal\ grafts}$$

To assess (B), achievement of successful transplantation of previously rejected donor liver following viability testing using NMLP, we would evaluate 90-day patient survival rate, as an indicator or liver function and/or viability following transplantation of marginal liver grafts following NMLP.

The 90-day patient survival rate will be calculated as the number of patients alive 90-day post NMLP treated marginal liver transplantation (numerator) divided by the total number of NMLP treated marginal liver transplants performed.

$$90\ day\ patient\ survival\ rate = \frac{Number\ of\ patients\ alive\ at\ 90\ day\ post\ transplantation}{Total\ number\ of\ transplanations\ performed}$$

For (A), all livers undergoing NMLP treatment will be included for evaluation in the interim and final analyses. For (B), all transplantations performed will be included for evaluation in the interim and final analyses. The rate outcomes will be reported together with confidence intervals using the Wilson (1927) method [20].

13.3.2 Secondary Analysis

For all secondary outcome measures, analyses will be mainly descriptive. Continuous exploratory measures will be summarised via means, medians, standard deviations and ranges. Categorical measures will be summarised with number and proportion in each category. To model repeated measures over time (e.g. quality of life), a linear mixed effects model (taking into account subject correlation) using parametric and more flexible models may be considered. Time to event outcomes will be assessed using the method of Kaplan and Meier. Median survival with corresponding 95% confidence interval will also be reported (where appropriate).

The assessment of graft function post-transplantation by incidence of primary non-function and early allograft dysfunction will be carried out by comparing results with a contemporary matched recipient group of patients obtained from a prospectively maintained database, with adjustment for potential confounders. The contemporary matched recipient group will be matched using the following:

- Patient Characteristics: Age, Sex, BMI, MELD, UKELD, Aetiology
- Donor Liver Characteristics: DCD or DBD, Sex.

13.3.3 Exploratory Analysis

Exploratory analysis on identification of novel biomarkers that are indicative of liver quality and function will be conducted. We will also explore how the trial results will compare with the contemporary data provided by the on-going NHSBT Liver Transplant audit data.

Such analyses may, by chance, generate false negative or positive results. Those carried out will be interpreted with caution and treated as hypothesis-generating.

13.3.4 Health Economic Analysis

More details can be found in Section 2.2.3 and 6.10. A separate analysis plan will be written before analysis is carried out.

13.4 Planned Sub Group Analyses

No subgroup analyses are planned.

13.5 Planned Interim Analysis

As we have utilised adaptive designs for both assessments of (A) feasibility of NMLP and (B) successful transplantation of rescued livers, there are planned formal interim assessments for both.

For (A), using a **Simon's two-stage design** [16] there is only one formal interim assessment.

Stage 1A: 24 marginal grafts will be perfused and assessed in the first stage. We would stop early and conclude that the NMLP procedure is infeasible at the end of the first stage if there are fewer than 8 recovered livers. Otherwise, proceed to Stage 2A to recruit up to 29 additional marginal grafts. Acceptance of donated marginal livers will not be halted while Stage 1A is assessed.

For (B) in assessing successful transplantation based on 90-day patient survival, the single arm optimal 3-stage design [17] included two interim assessments of accumulating data.

Stage 1B: Following transplantation in 3 patients, the trial will stop early (concluding P≤0.73) if there are fewer than 2 patients achieving 90-day survival. Otherwise an additional 8 transplantations will be performed.

Stage 2B occurs after transplantation in 11 patients (combined first and second stages) the trial will stop early (concluding P≤0.73) if there are seven or fewer successes, otherwise an additional 11 transplantations will be performed.

In general for both (A) and (B), recruitment will not be suspended whilst interim analyses are being performed and the DMC is reviewing the data. If recruitment is fast, speedy review will be necessary to ensure the utility of interim decisions.

To maximise patient safety for (B) following transplantation and discharge of the first 3 patients (stage 1B), recruitment will be paused to allow the DMC to assess the initial safety data. If the DMC considers the patients to be recovering well, with liver function that would be expected at this stage, recruitment can continue prior to the patients reaching the primary end-point of 90-day survival. A follow-on report will be sent to the DMC once the third patient reaches the primary end-point. For the second stage (transplantation of 11 patients), a report containing safety and any 90-day survival data will be supplied to the DMC for review and a meeting held after discharge of all 11 patients. Recruitment will not be paused at this point. A follow-on report will again be sent once the 11th patient reaches the primary endpoint (90-day survival).

13.6 Planned Final Analyses

For (A), the method will be only deemed successful if 22 or more donated marginal livers out of 53 treated with NMLP meet the viability criteria at the end of Stage 2A.
For (B), after Stage 3B, the method will only be deemed successful if at least 18 out of a total of 22 transplants are successful at the 90-day survival assessment.

14 TRIAL ORGANISATIONAL STRUCTURE

14.1 Sponsor

The University of Birmingham
Edgbaston
Birmingham
B15 2TT
United Kingdom

The University of Birmingham will act as single sponsor for all UK sites on receipt of written evidence of local national ethics and regulatory authority approval.

All serious adverse events occurring in the UK will be reported initially to the Central Trials Office (Birmingham, UK) for clinical evaluation and review, prior to reporting to the National ethics committees and competent authorities in the UK in accordance with country specific regulations.

14.2 Coordinating Centre

The trial is being conducted under the auspices of the Cancer Research UK Clinical Trials Unit (CRCTU), The University of Birmingham according to their local procedures.

14.3 Trial Management Group

The TMG will be responsible for the day-to-day running and management of the trial and will meet by teleconference or in person as required. Members of the TMG include the CI, Co-investigators, Project Manager, Trial Management Team Leader, Senior Trial Coordinator, Trial Coordinator, Lead Trial Statistician, and Trial Statistician. The TMG will have regular meetings during recruitment.

14.4 Trial Steering Committee

The TSC will provide overall supervision and representation for both the funders and sponsor of the study, whilst providing advice through its independent chair. Membership includes members of the TMG, selected Principal Investigators and where possible, consumer representatives. Other members/observers may be invited upon request. The ultimate decision for the continuation of the study lies with the TSC. The TSC will meet annually, or as requested by the TMG and DMC.

14.5 Data Monitoring Committee

The DMC will consist of independent members, including two independent clinicians with relevant expertise and an independent statistical expert.

Data analyses will be supplied in confidence to an independent DMC, which will be asked to give advice on whether the accumulated data from the trial, together with the results from other relevant research, justifies the continuing recruitment of further patients. The DMC will operate in accordance with a trial specific charter based upon the template created by the Damocles Group.

The DMC will meet at 2 scheduled time points. These meetings will take place after the 2 interim analysis time points. Interim analysis is planned after 3 patients have been transplanted and have reached the primary endpoint of 90 day survival, and after a further 8 patients (11 patients in total) have been transplanted and the 11th patient has been discharged from hospital. For the first interim safety analysis point, the first three patients' data will be reviewed after their hospital discharge. If the DMC consider that the patients are recovering well and have liver function that would be expected at that stage, recruitment will continue prior to the third patient reaching the primary endpoint of 90-day survival. The DMC will review the data again when the third patient reaches the 90-day endpoint, and can convene a meeting if required. For the second stage, following the discharge of the 11th patient transplant, a report containing safety and any 90-day survival data will be supplied to the DMC for review and a meeting held. A follow-on report will again be sent once the 11th patient reaches the primary endpoint (90-day survival), and if requested a meeting will be convened.

Additional meetings may be called if recruitment is much faster than anticipated and the DMC may, at their discretion, request to meet more frequently or continue to meet following completion of recruitment. An emergency meeting may also be convened if a safety issue is identified. The DMC will report directly to both the VITTAL Trial Management Group (Chief Investigator) who will convey the findings of the DMC to the TSC and funders/sponsor as appropriate or when specifically requested by these parties.

The DMC may consider recommending the discontinuation of the trial if the recruitment rate or data quality are unacceptable or if any issues are identified which may compromise patient safety. The trial could also be stopped early if the interim analyses showed undesirable safety issues or differences between treatments that were deemed to be convincing to the clinical community.

15 FINANCE

This is a clinician-initiated and clinician-led trial funded by the Wellcome Trust under a project award entitled "Viability testing and transplantation of marginal livers'.

In addition OrganOx Ltd (a named collaborator on the above award application) have provided the OrganOx *metra*™ device comprising of 1 retained unit and up to 53 disposable sets.

No individual per patient payment will be made to NHS Trusts, Investigators or patients.

16 ETHICAL AND REGULATORY CONSIDERATIONS

The trial will be performed in accordance with the recommendations guiding physicians in biomedical research involving human subjects, adopted by the 18th World Medical Association General Assembly, Helsinki, Finland, June 1964, amended at the 48th World Medical Association General Assembly, Somerset West, Republic of South Africa, October 1996 (website: http://www.wma.net/en/30publications/10policies/b3/index.html).

The trial will be conducted in accordance with the Research Governance Framework for Health and Social Care, the applicable UK Statutory Instruments, (which include the Medicines for Human Use Clinical Trials 2004 and subsequent amendments and the Data Protection Act 1998 and Human Tissue Act 2008 and the Guidelines for Good Clinical Practice (GCP). This trial will be carried out under a Clinical Trial Authorisation in accordance with the Medicines for Human Use Clinical Trials regulations. The protocol will be submitted to and approved by the main Research Ethics Committee (REC) prior to circulation.

Before any patients are enrolled into the trial, the Principal Investigator at each site is required to obtain local R&D approval. Sites will not be permitted to enrol patients until written confirmation of R&D approval is received by the Trials Office.

It is the responsibility of the Principal Investigator to ensure that all subsequent amendments gain the necessary local approval. This does not affect the individual clinicians' responsibility to take immediate action if thought necessary to protect the health and interest of individual patients.

The Investigator will ensure that this trial is conducted in full conformity with:

- European Commission Medical Device Guidelines relating to the application of the EU Directives on Medical Devices
- Guide to European Medical Device Trials and BS EN ISO 14155

17 CONFIDENTIALITY AND DATA PROTECTION

Personal data recorded on all documents will be regarded as strictly confidential and will be handled and stored in accordance with the General Data Protection Regulation and the Data Protection Act (2018). With the patient's consent, their full name, date of birth, hospital number will be collected at trial entry to formally identify the patient for internal/external (on-site) monitoring and study audit purposes by representatives from either the study sponsor, NHS trust and national regulatory bodies.

Patients will be identified using only their unique registration number, patient initials on the Case Report Form and correspondence between the Trials Office and the participating site. In addition the patients are requested to give permission for the Trials Office to be sent a copy of their signed Informed Consent Form which will not be anonymised. This will be used to perform in-house monitoring of the consent process.

The Investigator must maintain documents not for submission to the Trials Office (e.g. Patient Identification Logs) in strict confidence. In the case of specific issues and/or queries from the regulatory authorities, it will be necessary to have access to the complete trial records, provided that patient confidentiality is protected.

The Trials Office will maintain the confidentiality of all patient's data and will not disclose information by which patients may be identified to any third party. Representatives of the VITTAL trial team may be required to have access to patient's notes for quality assurance purposes but patients should be reassured that their confidentiality will be respected at all times.

18 INSURANCE AND INDEMNITY

The University of Birmingham employees are indemnified by the University insurers for negligent harm caused by the design or co-ordination of the clinical trials they undertake whilst in the University's employment.

In terms of liability at a site, NHS Trust and non-Trust hospitals have a duty to care for patients treated, whether or not the patient is taking part in a clinical trial. Compensation is therefore available via NHS indemnity in the event of clinical negligence having been proven.

The University of Birmingham cannot offer indemnity for non-negligent harm. The University of Birmingham is independent of any pharmaceutical company, and as such it is not covered by the Association of the British Pharmaceutical Industry (ABPI) guidelines for patient compensation.

19 PUBLICATION POLICY

Results of this trial will be submitted for publication in a peer reviewed journal. The manuscript will be prepared by the TMG and authorship will be determined by mutual agreement.

Any secondary publications and presentations prepared by Investigators must be reviewed by the TMG. Manuscripts must be submitted to the TMG in a timely fashion and in advance of being submitted for publication, to allow time for review and resolution of any outstanding issues. Authors must acknowledge that the trial was performed with the support of University of Birmingham. Intellectual property rights will be addressed in the Clinical Study Site Agreement between Sponsor and site.

20 REFERENCE LIST

REFERENCES

1. Williams R, Aspinall R, Bellis M, Camps-Walsh G, Cramp M, Dhawan A, et al. Addressing liver disease in the UK: a blueprint for attaining excellence in health care and reducing premature mortality from lifestyle issues of excess consumption of alcohol, obesity, and viral hepatitis. Lancet. 2014;384(9958):1953-97. doi: 10.1016/S0140-6736(14)61838-9. PubMed PMID: 25433429.

2. Williams R, Ashton K, Aspinall R, Bellis MA, Bosanquet J, Cramp ME, et al. Implementation of the Lancet Standing Commission on Liver Disease in the UK. Lancet. 2015;386(10008):2098-111. doi: 10.1016/S0140-6736(15)00680-7. PubMed PMID: 26700394.

3. European Liver Transplant Registry. Available from: http://www.eltr.org.

4. Organ Donation and Transplantation: Activity Report 2014/15. Available from: http://www.odt.nhs.uk. Retrieved October 30, 2015, from http://nhsbtmediaservices.blob.core.windows.net/organ-donation-assets/pdfs/activity_report_2014_15.pdf.

5. Sotiropoulos GC, Radtke A, Molmenti EP, Schroeder T, Baba HA, Frilling A, et al. Long-term follow-up after right hepatectomy for adult living donation and attitudes toward the procedure. Ann Surg. 2011;254(5):694-700; discussion -1. doi: 10.1097/SLA.0b013e31823594ae. PubMed PMID: 22005145.

6. Simoes P, Kesar V, Ahmad J. Spectrum of biliary complications following live donor liver transplantation. World journal of hepatology. 2015;7(14):1856-65. doi: 10.4254/wjh.v7.i14.1856. PubMed PMID: 26207167; PubMed Central PMCID: PMCPMC4506943.

7. Mirza DF, Gunson BK, Da Silva RF, Mayer AD, Buckels JA, McMaster P. Policies in Europe on "marginal quality" donor livers. Lancet. 1994;344(8935):1480-3. PubMed PMID: 7968124.

8. National standards for organ retrieval from deceased donors (joint with NHSBT). Available from: http://www.bts.org.uk. Retrieved September 1, 2015, from http://www.bts.org.uk/Documents/9.1.13 Retrieval Standards Document v2 6 effective 010113.pdf.

9. Feng S, Goodrich NP, Bragg-Gresham JL, Dykstra DM, Punch JD, DebRoy MA, et al. Characteristics associated with liver graft failure: the concept of a donor risk index. American journal of transplantation : official journal of the American Society of Transplantation and the American Society of Transplant Surgeons. 2006;6(4):783-90.

10. Braat AE, Blok JJ, Putter H, Adam R, Burroughs AK, Rahmel AO, et al. The Eurotransplant donor risk index in liver transplantation: ET-DRI. Am J Transplant. 2012;12(10):2789-96. doi: 10.1111/j.1600-6143.2012.04195.x. PubMed PMID: 22823098.

11. Belzer FO, Southard JH. Principles of solid-organ preservation by cold storage. Transplantation. 1988;45(4):673-6.

12. Oniscu GC, Randle LV, Muiesan P, Butler AJ, Currie IS, Perera MT, et al. In situ normothermic regional perfusion for controlled donation after circulatory death-the United kingdom experience. Am J Transplant. 2014;14(12):2846-54. doi: 10.1111/ajt.12927. PubMed PMID: 25283987.

13. Ravikumar R, Jassem W, Mergental H, Heaton N, Mirza D, Perera MT, et al. Liver transplantation after ex vivo normothermic machine preservation: a Phase 1 (first-in-man) clinical trial. Am J Transplant. 2016. doi: 10.1111/ajt.13708. PubMed PMID: 26752191.

14. Perera M, Mergental H, Stephenson B, Roll GR, Cilliers H, Liang R, et al. First human liver transplantation using a marginal allograft resuscitated by normothermic machine perfusion. Liver transplantation : official publication of the American Association for the Study of Liver Diseases and the International Liver Transplantation Society. 2015. doi: 10.1002/lt.24369. PubMed PMID: 26566737.

15. Dindo D, Demartines N, Clavien PA. Classification of surgical complications: a new proposal with evaluation in a cohort of 6336 patients and results of a survey. Ann Surg. 2004;240(2):205-13. Epub 2004/07/27. PubMed PMID: 15273542; PubMed Central PMCID: PMC1360123.

16. Simon R. Optimal two-stage designs for phase II clinical trials. Control Clin Trials. 1989;10(1):1-10. PubMed PMID: 2702835.

17. Chen TT. Optimal three-stage designs for phase II cancer clinical trials. Statistics in medicine. 1997;16(23):2701-11. PubMed PMID: 9421870.

18. NHSBT. Activity report 2014/15. 2014/15.

19. Dutkowski P, Schlegel A, Slankamenac K, Oberkofler CE, Adam R, Burroughs AK, et al. The use of fatty liver grafts in modern allocation systems: risk assessment by the balance of risk (BAR) score. Ann Surg. 2012;256(5):861-8; discussion 8-9. doi: 10.1097/SLA.0b013e318272dea2. PubMed PMID: 23095632.

20. Wilson EB. Probable Inference, the Law of Succession, and Statistical Inference. . Journal of the American Statistical Association. 1927;22(158):209-12.

APPENDIX 1 – CLAVIEN-DINDO CLASSIFICATION OF SURGICAL COMPLICATIONS

Grade	Definition
**I**	Any deviation from the normal postoperative course without the need for pharmacological treatment or surgical, endoscopic and radiological interventions.
**II**	Requiring pharmacological treatment with drugs other than such allowed for grade I complications. Blood transfusions and total parenteral nutrition are also included.
**III**	Requiring surgical, endoscopic or radiological intervention.
**IIIa**	Intervention not under general anaesthesia.
**IIIb**	Intervention under general anaesthesia.
**IV**	Life-threatening complications (including CNS complications) requiring HDU/ITU management.
**IVa**	Single organ dysfunction (including dialysis).
**IVb**	Multi-organ dysfunction.
**V**	Death of a patient.
**Suffix ‘d’**	If the patient suffers from a complication at the time of discharge, the suffix ‘d’ (for disability) is added to the respective grade of complication. This label indicates the need for a follow-up to fully evaluate the complication.

APPENDIX 2 – EQ-5D-5L

EQ-5D-5L

Sample

Health Questionnaire

English version for the UK

UK (English) © 2009 EuroQol Group EQ-5D™ is a trade mark of the EuroQol Group

Under each heading, please tick the ONE box that best describes your health TODAY.

MOBILITY

I have no problems in walking about	☐
I have slight problems in walking about	☐
I have moderate problems in walking about	☐
I have severe problems in walking about	☐
I am unable to walk about	☐

SELF-CARE

I have no problems washing or dressing myself	☐
I have slight problems washing or dressing myself	☐
I have moderate problems washing or dressing myself	☐
I have severe problems washing or dressing myself	☐
I am unable to wash or dress myself	☐

USUAL ACTIVITIES *(e.g. work, study, housework, family or leisure activities)*

I have no problems doing my usual activities	☐
I have slight problems doing my usual activities	☐
I have moderate problems doing my usual activities	☐
I have severe problems doing my usual activities	☐
I am unable to do my usual activities	☐

PAIN / DISCOMFORT

I have no pain or discomfort	☐
I have slight pain or discomfort	☐
I have moderate pain or discomfort	☐
I have severe pain or discomfort	☐
I have extreme pain or discomfort	☐

ANXIETY / DEPRESSION

I am not anxious or depressed	☐
I am slightly anxious or depressed	☐
I am moderately anxious or depressed	☐
I am severely anxious or depressed	☐
I am extremely anxious or depressed	☐

UK (English) © 2009 EuroQol Group EQ-5D™ is a trade mark of the EuroQol Group

Sample

- We would like to know how good or bad your health is TODAY.
- This scale is numbered from 0 to 100.
- 100 means the <u>best</u> health you can imagine.
  0 means the <u>worst</u> health you can imagine.
- Mark an X on the scale to indicate how your health is TODAY.
- Now, please write the number you marked on the scale in the box below.

YOUR HEALTH TODAY = [ ]

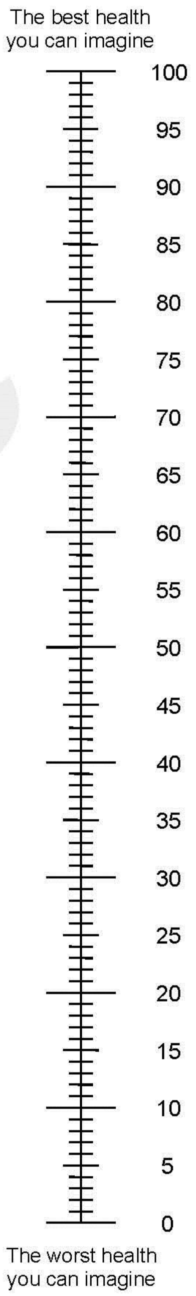

Sample

UK (English) © 2009 EuroQol Group EQ-5D™ is a trade mark of the EuroQol Group

APPENDIX 3 – LIST OF UK TRANSPLANT CENTRES

London – Royal Free Hospital
London – King's College Hospital
Birmingham – Queen Elizabeth Hospital
Leeds – St James's University Hospital
Newcastle – Freeman Hospital
Cambridge – Addenbrooke's Hospital
Edinburgh – Royal Infirmary

APPENDIX 4 – DONOR RISK INDEX

https://gastro.cchmc.org/calculators/donor-risk-index/

APPENDIX 5 – BAR SCORE

http://www.assessurgery.com/bar-score/bar-score-calculator/

APPENDIX 6 – BIOLOGICAL SPECIMENS

Biological Specimens

All specimens collected as part of the recipient's routine clinical care, such as postoperative blood samples for routine laboratory analysis, will be analysed and stored locally as per normal local procedure. Results required by the trial protocol will be uploaded to the online data collection system.

Study specific samples will be retained by the University of Birmingham.

These samples include:

- Perfusate samples from the perfusion device
- Patient plasma, serum and urine samples as specified, other than those required for routine clinical care.
- Pre- and post-perfusion liver and bile duct biopsies
- Bile

Provision will be made on the study consent form and ethics applications for storage and later use of these samples for ancillary studies.

Time Point	Description	Code
**Visit 1**		
**Admission**	Biochemistry<br>Haematology<br>Clotting	
**Post-induction (recipient samples)**	Serum	RS-0
	Plasma	RP-0
	MN Cells	RMC-0
	Urine	RU-0
**Pre-perfusion (Back Bench)**	Menghini Liver	L1<br>• Formalin<br>• Frozen<br>EM
	Common bile duct	CBD1<br>Formalin
**Pre-perfusion (OrganOx)**	Perfusate<br>5ml spun, filtered and aliquoted	P-0
	Perfusate gas analysis	APG-0<br>VPG-0
**Throughout perfusion**	Perfusate<br>5ml spun, filtered and aliquoted	P-0.25<br>P-0.50<br>P-0.75<br>P-1.0<br>P-x (then every hour)<br>P-end
	Perfusate gas analysis	APG-x<br>VPG-x (every 30 mins for first 4 hours and every hour thereafter)

	Bile<br>Up to 3 cryovials every 2 hours	B-2.0<br>B-4.0<br>B-x
**At end of perfusion**	Perfusate cultures for microbiology<br>30ml, 10ml in a sterile universal container and in each culture bottle	P-culture
**At end of perfusion**	Perfusate cells	P-cells
**After 4 hours of perfusion (transplant decision)**	Menghini Liver	L2<br>• Formalin<br>If perfusion is going to last less than 6 hours…<br>• Frozen<br>EM
**End of perfusion (if lasting longer than 6 hours)**	Menghini Liver	L3<br>• Formalin<br>• Frozen<br>EM
**Transplant**		
**15 minutes after portal or arterial reperfusion**	Biochemistry<br>Haematology<br>Clotting	
	Serum	RS-T
	Plasma	RP-T
	MN Cells	RMC-T
	Urine	RU-T
**Before bile duct anastamosis**	Menghini Liver	L4<br>• Formalin<br>• Frozen<br>EM
	Common bile duct	CBD2<br>Formalin
**Post-op**		
**Days 1-7**	Biochemistry<br>Haematology<br>Clotting	
**Day 4 (+/-2 days)**	Serum	RS-4
	Plasma	RP-4
	MN Cells	RMC-4
	Urine	RU-4
**Visit 2**		
**Day 30 - OPD**	Biochemistry<br>Haematology<br>Clotting	
	Serum	RS-30
	Plasma	RP-30
	MN Cells	RMC-30
**Visit 3**		
**Day 90 - OPD**	Biochemistry<br>Haematology<br>Clotting	
	Serum	RS-90

	Plasma	RP-90
	MN Cells	RMC-90
**Visit 4**		
**Day 180 - OPD**	Biochemistry<br>Haematology<br>Clotting	
	Serum	RS-180
	Plasma	RP-180
	MN Cells	RMC-180

Regulatory aspects

All samples will be collected in accordance with national regulations and requirements including standard operating procedures for logistics and infrastructure. Samples will be taken in appropriately licensed premises, stored and transported in accordance with the HTA guidelines and trust policies. Samples for long-term storage will be kept at The University of Birmingham.

Recipient blood samples

Blood results from samples taken as part of the standard of care will be recorded on days 1-7, and on follow-up visits on day 30, 90 and 180. In addition to these bloods, extra samples (2 x CPT 8 ml tubes and 1 x SST 10ml tube) will be taken on day 4 (+/- 2days), and on each follow-up visit and processed as described below.

At each time-point where recipient blood is collected

- Samples of whole blood will be sent for FBC, U+E, LFT's and clotting (standard of care)
- 2x CPT 8 ml tubes will be obtained for plasma and mononuclear cells
- 1x SST 10ml tube will be obtained for serum

To ensure minimal sample degradation and pre-analytical variability, whole blood should be kept at room temperature prior to separation of plasma from cellular parts. Separation of cells from serum should be achieved by centrifugation at 1500g for 10 min at room temperature as close as possible to blood collection. CPT Tubes are prepared by centrifugation at 1500g for 20 minutes after which plasma and mononuclear cells can be processed accordingly. Standard Operating Procedures will reflect practical time points for the handling and processing of samples. After centrifugation plasma and serum samples will be stored at -80 ^{o}C.

Perfusion fluid (perfusate) samples

Perfusate samples will be collected at time-points described during NMLP. 5ml sample will be aliquoted into Eppendorf tubes and centrifuged in a Hettich Mikro Microfuge at 15000g for 20 seconds. The supernatant will be decanted into 5x1ml aliquots in cryovials and either stored at -80 ^{o}C or kept at 4 ^{o}C for early processing depending on tests required.

Biopsies

Biopsy segments will be divided into two longer segments and 1mm length piece of tissue.

The longer segments will be stored in pre-prepared formalin and prepared to enable analysis. The smaller 1mm tissue sample will be stored in glutaraldehyde for electron microscopy. The 3rd menghini biopsy (at end of perfusion) is only required in cases where the perfusion lasts longer than 6 hours (i.e. longer than 2 hours after the 4hr biopsy). This is to give the clearest picture of liver histology prior to transplant.

APPENDIX 7 – DEFINITION OF ADVERSE EVENTS

Adverse Event (AE)
Any untoward medical occurrence in a subject.
NOTE this definition does not imply that there is a relationship between the adverse event and the device under investigation.

Serious Adverse Event (SAE)
An adverse event that
a) led to a death,
b) led to a serious deterioration in the health of the subject that
1) resulted in a life-threatening illness or injury,
2) resulted in a permanent impairment of a body structure or a body function,
3) required in-patient hospitalization or prolongation of existing hospitalization,
4) resulted in medical or surgical intervention to prevent permanent impairment to body structure or a body function.
c) led to foetal distress, foetal death or a congenital abnormality or birth defect.

Adverse Device Effect (ADE)
Any untoward and unintended response to a medical device.
NOTE 1 This definition includes any event resulting from insufficiencies or inadequacies in the instructions for use or the deployment of the device.

Serious Adverse Device Effect (SADE)

Adverse device effect that has resulted in any of the consequences characteristic of a serious adverse event or that might have led to any of these consequences if suitable action had not been taken or intervention had not been made or if circumstances had been less opportune.

Unanticipated Serious Adverse Device Effects (USADE)

Any serious adverse device effect which, by its nature, incidence, severity or outcome, has not been identified in the anticipated AE’s listed in section 8.1.1.

Device Deficiency

Inadequacy of a medical device with respect to its identity, quality, durability, reliability, safety or performance. Device deficiencies include malfunctions, use errors and inadequate labelling.

Use error

Act or omission of an act that results in a different medical device response than intended by the manufacturer or expected by the user. Use error includes slips, lapses and mistakes. An unexpected physiological response of the subject does not itself constitute a use error.

Severity definitions

The following definitions will be used to determine the severity rating for all adverse events:

Mild: awareness of signs or symptoms, that does not interfere with the subject's usual activity or is transient that resolved without treatment and with no sequelae.

Moderate: a sign or symptom, which interferes with the subject's usual activity.

Severe: incapacity with inability to do work or perform usual activities.

APPENDIX 8 – WMA DECLARATION OF HELSINKI

At the time of writing the 1996 version is the legally required version in the EU; see link to shared drive: S:\General\Reference documents\WMA Declaration of Helsinki version 1996.docx

VITTAL

Statistical Analysis Plan

An open label, non-randomised, prospective, single arm trial, using normothermic machine liver perfusion NMLP to test viability and transplantation of marginal livers

Version:

V 2.0 2nd August 2018

Sponsor: **The University of Birmingham**

Coordinating Centre: *CRCTU Birmingham*

EUDAMED number: **CIV-GB-16-08-016567**

Author(s):

Name: Amanda Kirkham **Signature:**

Trial role: Trial Statistician

Date: 2nd August 2018

Reviewed and approved by:

Name: Dr Christina Yap **Signature:**

Trial role: Lead Biostatistician and Co-investigator

Date:

Name: Professor Darius Mirza **Signature:**

Trial role: Chief Investigator

Date:

Cancer Research UK Clinical Trials Unit

@CRCTU

Key personnel involved in the Statistical Analysis Plan:

Name	Trial role
Professor Darius Mirza	Chief Investigator
Dr Christina Yap	Lead Statistician & Co-investigator
Manpreet Wilkhu	Trial Coordinator
Amanda Kirkham	Trial Statistician

Document Control Sheet

Statistical Analysis Plan version:	Reason for update:
v0.1 10th March 2017	Initial draft
v0.2 22nd March 2017	Amendments to v0.1
V1.0 29th March 2017	Version 1.0
V 2.0 2nd August 2018	Minor Amendments (and formatting) to version 1.0, updated to be in line with Protocol v2.0b 12-Jul-2017 :
	• 1A.3.2: typo correction "(see section A)" changed to "(see section B)"
	• "(e.g. Wilson method)" changed to "(Wilson (1927) method)" throughout with reference added
	• (1A.11, 1B.8) Clarify that secondary and subgroup analyses will be mainly descriptive.
	• 1B.8:; "for objective one" removed; "will" changed to "may";; list of specific liver function tests given; "descriptively" removed; "will" changed to "may only"

CONTENTS

Section	Contents	Page
**I**	**Trial Introduction**	
II	Purpose of the Statistical Analysis Plan	5
III	Summary of the Trial	5
IV	Trial Schema	7
**1A**	**Feasibility of NMLP on Marginal Livers Analyses**	
1A.1	Timing and Reporting of Interim and Final Analyses	8
1A.2	Recruitment and Randomisation	8
1A.2.1	Recruitment	8
1A.2.2	Randomisation	8
1A.2.3	Ineligible Patients./Grafts	8
1A.3	Data Quality	8
1A.3.1	CRF Return Rate	8
1A.3.2	Length of Follow-up	9
1A.4	Trial Populations	9
1A.4.1	Baseline Characteristics	9
1A.4.2	Definition(s) of Populations for Analysis	9
1A.5	Treatment Received	9
1A.6	Toxicity and Safety Analysis	9
1A.7	Analysis of Outcome Measures	10
1A.7.1	Definition and Calculation of Primary Outcome Measures	10
1A.7.2	Descriptive Analyses for the Primary Outcome Measures	10
1A.7.3	Hypothesis Testing for the Primary Outcome Measures	10
1A.7.3.1	Hypotheses Under Test	10
1A.7.3.2	Methods	10
1A.7.3.3	Sample Size Calculations	10
1A.8	Hypothesis Testing for Secondary Outcome Measures	10
1A.9	Additional Analyses – Exploratory Outcomes	11
1A.10	Decision Criteria	11
1A.11	Subgroup Analyses	11
**1B**	**Transplantation of Rejected Livers Following Viability Testing Using NMLP**	
1B.1	Timing and Reporting of Interim and Final Analyses	12
1B.2	Recruitment and Randomisation	12
1B.2.1	Recruitment	12
1B.2.2	Randomisation	12
1B.2.3	Ineligible Patients	12
1B.3	Data Quality	12
1B.4	Trial Populations	13
1B.4.1	Baseline Characteristics	13
1B.4.2	Definition(s) of Populations for Analysis	13
1B.5	Treatment Received	13
1B.6	Toxicity and Safety Analysis	13
1B.7	Analysis of Outcome Measures	14
1B.7.1	Definition and Calculation of Primary Outcome Measures	14
1B.7.2	Descriptive Analyses for the Primary Outcome Measures	14
1B.7.3	Hypothesis Testing for the Primary Outcome Measures	14
1B.7.3.1	Hypotheses Under Test	14
1B.7.3.2	Methods	14
1B.7.3.3	Sample Size Calculations	15
1B.8	Hypothesis Testing for Secondary Outcome Measures	15
1B.9	Additional Analyses and Exploratory Outcomes	15

Section	Contents	Page
1B.10	Decision Criteria	16
1B.11	Subgroup Analyses	17
**2**	**Further Analysis Information**	
2.1	Health Economics	18
2.2	Statistical Software	18
2.3	Storage and Archiving	18
2.4	References	18

I TRIAL INTRODUCTION

For background information please refer to the trial protocol background section.

II Purpose of the Statistical Analysis Plan

This Statistical Analysis Plan (SAP) provides guidelines for the analysis and presentation of results for the VITTAL trial. This plan, along with all other documents relating to the analysis of this trial, will be stored in the 'Statistical Documentation' section of the Trial Master File. The statistical analysis will be carried out by the Trial Statistician.

III Summary of the Trial

TRIAL SYNOPSIS

**Title**	Viability testing and transplantation of marginal livers.<br>An open label, non-randomised, prospective, single arm trial, using normothermic machine liver perfusion NMLP to test viability and transplantation of marginal livers	
**Trial Design**	The proposed design is an open label, non-randomised, prospective, single arm trial, performing normothermic machine liver perfusion (NMLP) on rejected livers until we reach 22 clinical transplants. It is designed with two linked components to assess;<br>• Feasibility of NMLP in rejected organ recovery using an established 2-stage design (Simon, 1989)<br>• Successful transplantation using an optimal 3-stage design (Chen, 1997).	
**Trial duration**	It is anticipated that recruitment will take 15 months. All patients will be followed up for 6 months post-transplant. This recruitment is based on an average of 1-2 patients being recruited each month from the Queen Elizabeth Hospital, Birmingham, UK	
**Objectives and Outcome Measures**		
**Co-Primary**	**Objective**	**Outcome Measure**
	1a Establish the feasibility of NMLP as a means to increase the number of transplantable livers	1a "Rescue rate" i.e. the number of rejected livers that can be salvaged and used for clinical transplantation
	1b Achievement of successful transplantation using rejected liver grafts	1b 90-day patient survival
**Secondary**	**Objective**	**Outcome Measure**
	Assess the liver graft function following transplantation (by incidence of primary non-function, and early allograft dysfunction)	• Liver function tests<br>• 90-day graft survival<br>• 12-month patient and graft survival<br>• The secondary endpoints and other outcome measures will be compared with a contemporary matched recipient group

	Assess the physiological response to reperfusion of the perfused grafts	Post-reperfusion syndrome (Defined as a decrease in mean arterial pressure (MAP) of more than 30% from the baseline value for more than one minute during the first five minutes after reperfusion (assessed in the context of inotrope use)).
	To compare clinical course of transplantation using rejected liver grafts with historical controls carried out with 'transplantable' liver grafts	– Adverse event rates and severity, graded according to the Clavien-Dindo classification<br>– Primary graft non-function rate<br>– 90-day graft survival<br>– 12-month patient and graft survival<br>– Incidence of non-anastomotic biliary strictures<br>– Requirement of renal replacement therapy
	Identify impact upon quality of life after transplantation with these liver grafts	– Quality of life analysis using EQ-5D-5L questionnaire
**Exploratory**	**Objective**	**Outcome Measure**
	Identify novel biomarkers that are indicative of liver quality and function	This will be performed through careful analysis of biological samples and through correlation of findings with perfusion outcome and if transplanted, clinical outcome
	Identify the feasibility of using NMLP to increase the number of liver transplants	Health economic analysis

IV Trial Schema

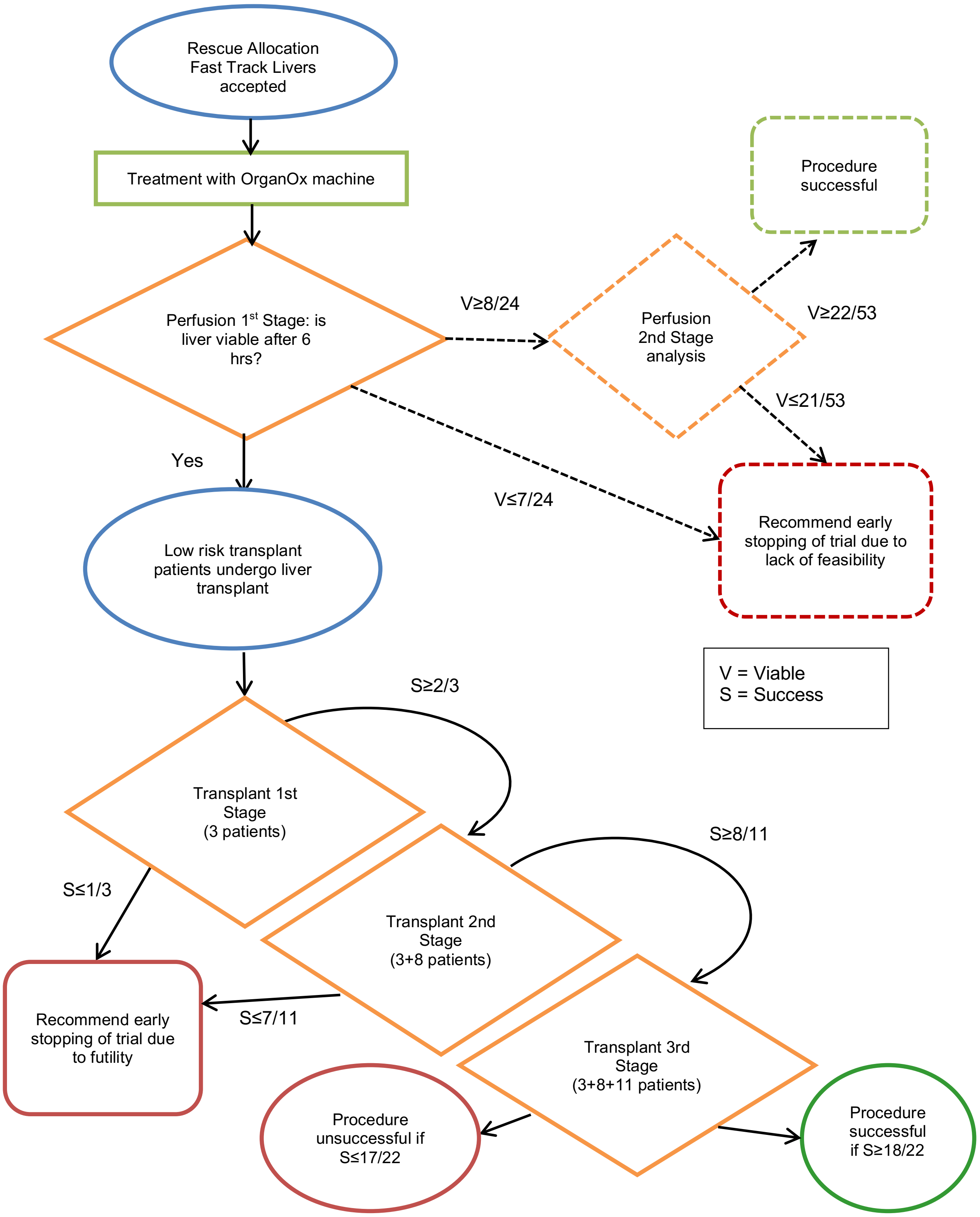

Figure 1: Trial Design

SECTION 1A: FEASIBILITY OF NMLP ON MARGINAL LIVERS

1A.1: TIMING AND REPORTING OF INTERIM AND FINAL ANALYSES

There is only one interim assessment which will occur after 24 marginal liver grafts have been perfused. The final assessment point will be reached when 53 marginal livers have been perfused. Assessment of the livers undergoing NMLP will be assessed for the rescue rate primary end-point after receiving NMLP.

1A.2: RECRUITMENT AND RANDOMISATION

1A.2.1 Recruitment

The analysis will include:

- Date the snapshot was taken
- Dates when the trial opened and closed for recruitment
- Recruitment over time (monthly or quarterly) and an average monthly recruitment rate for patients

1A.2.2 Randomisation

There is no randomisation in the VITTAL trial.

1A.2.3 Ineligible Patients/Grafts

No patients are directly involved in this part of the trial, however all rejected donated livers meeting acceptability criteria will be eligible for the NMLP feasibility part of the trial if a suitable transplant recipient is registered on the VITTAL trial. Graft inclusion and exclusion criteria can be found in the Trial Synopsis in the Protocol.

1A.3: DATA QUALITY

Completion rates for all CRFs and outcome variables will be analysed as part of statistical data validation as set out in the Data Validation Plan (DVP); any missing data/forms will be flagged and reported to the Trial Coordinator (TC).

1A.3.1 CRF Return Rate

- The number of CRFs returned against the number expected at that time point in in the trial will be reported
- The amount of missing data and the relevant fields on the CRF will be reported if it is found that there is substantial data missing

1A.3.2 Length of patient follow-up

- Whilst the end-point for trial participation will be 6 months, patients undergoing transplantation will be followed up for a total of 12 and 24 months post transplantation
- Due to the nature of the trial it is not envisaged that patients will be lost to follow up, however, any patients that are lost to follow-up will not be excluded from the analysis but will be censored at the appropriate date

1A.3.2 Length of follow-up

Donated livers will be monitored throughout the perfusion stage (maximum time of 24 hours). Livers meeting transplantation criteria will be transplanted into patient recipients and will be followed up during the transplantation part of the trial (see sectionB). Livers that do not meet transplantation criteria will have sample biopsies taken and continue into Work Package 2.

1A.4: TRIAL POPULATIONS

1A.4.1 Baseline Characteristics

All collected donor characteristics will be presented: for continuous measures the mean (or median), standard deviation, minimum, maximum and IQR will be presented; for categorical variables the number and proportion per category will be presented. Appropriate plots will be included where required, especially for describing trends/changes over time.

The following donor demographics will be reported descriptively:

- Age
- Sex
- Ethnicity
- Cause of death
- Type of donor (DBD, DCD)
- Donor height
- Diabetes (UK-DRI)
- Smoking (UK-DRI)
- Donor risk index (DRI)
- Last and peak serum ALT/AST
- Last and peak serum sodium
- Last and peak GGT
- Bilirubin (used for UK-DRI)
- Albumin (for UK-DRI)
- Length of ITU (intensive therapy unit) stay
- BMI

1A.4.2 Definition(s) of Populations for Analysis

Any donated liver eligible and registered onto the trial will be included in the analysis population.

1A.5: TREATMENT RECEIVED

All trial registered livers will undergo the same NMLP technique as stated in Protocol sections 6 and 7.

The number of livers receiving NMLP will be reported, along with treatments received, concomitant and pre/post treatments. Any deviations from protocol will also be reported and will include reasons why.

1A.6: TOXICITY AND SAFETY ANALYSIS

This section relates to the safety and functioning of the normothermic perfusion machine. All adverse device effects (ADEs), serious adverse device effects (SADEs), unanticipated serious adverse device effects (USADEs), device deficiencies, and abnormal laboratory findings (e.g. positive microbiology results in perfusate) will be reported by number of occurrence, proportion, grade, type and classification. Refer to Protocol section 7 and Appendix 7.

1A.7: ANALYSES OF OUTCOME MEASURES

1A.7.1 Definition and Calculation of Primary Outcome Measures

The “Rescue rate” will be deemed successful if at end of trial ≥22/53 perfused grafts fulfil the acceptance criteria and go on to be transplanted.

Calculated using:

$$Rescue\ Rate = \frac{Number\ of\ viable\ perfused\ marginal\ grafts}{Total\ number\ of\ perfused\ marginal\ grafts}$$

1A.7.2 Descriptive Analyses for the Primary Outcome Measures

- The Rescue rate will be reported as a percentage, together with a 95% confidence interval (Wilson (1927) method)
- Descriptive statistics will present donated liver demographics
- Descriptive statistics will present safety data relating to SAP section B6
- The mean, median, minimum, maximum and IQR will be reported for continuous variable data where appropriate
- Number (count), proportion and percentage will be reported for categorical variable data

1A.7.3 Hypothesis Testing for the Primary Outcome Measures

1A.7.3.1 Hypotheses Under Test

- Null hypothesis is that the organ recovery rate is less than or equal to 30%
- Alternate hypothesis is that organ recovery rate is at least 50%

1A.7.3.2 Methods

- **Stage 1A of accrual:** 24 marginal grafts will be perfused and assessed at the first stage. Using the hypothesis from section 1A.7.3.1, we would reject the perfusion method at the end of the first stage of accrual if 7 or fewer livers were recovered. Otherwise, the trial proceeds into stage 2A.

- **Stage 2A of accrual:** Up to an additional 29 perfused marginal grafts will be accrued. We would accept the perfusion method as successful if 22 or more livers are recovered from the total of 53 marginal livers perfused.

1A.7.3.3 Sample Size Calculations

The feasibility of NMLP study has been designed as a Simon’s two-stage design (Simon, 1989) testing a null hypothesis that the rescue rate is 30% against an alternative hypothesis that it is 50%, with 90% power and 5% alpha. The total sample size requires 53 marginal livers to be perfused.

1A.8 Hypothesis Testing for Secondary Outcome Measures

No hypothesis testing is planned for secondary outcome measures.

1A.9 Additional Analyses – Exploratory Outcomes

Exploratory outcomes will form part of Work Package 2, these data will not be analysed by the trial statistician.

1A.10 Decision Criteria

Using a Simon's two-stage trial design (Simon, 1989):

Stage 1A of accrual: 24 marginal grafts will be perfused and assessed in the first stage. The trial will stop if there are 7 or fewer recovered livers. Otherwise proceed to stage 2A.

Stage 2A of accrual: Up to additional 29 marginal grafts will be perfused. The trial will stop of there are 21 or less recovered livers out of 53 perfused livers. We would accept the procedure as feasible if there are at least 22 recovered livers out of 53 perfused livers.

1A.11 Subgroup Analysis

The trial is not powered to perform statistical subgroup analyses however the following characteristics will be explored, reporting the numbers and percentages:

- DBD donated livers vs DCD donated livers
- Change in lactate in transplanted vs non-transplanted livers
- Bile production vs non-bile production
- Viable livers vs non-viable livers

SECTION 1B: TRANSPLANTATION OF REJECTED LIVERS FOLLOWING VIABILITY TESTING USING NMLP

1B.1: TIMING AND REPORTING OF INTERIM AND FINAL ANALYSES

Two interim assessment points are incorporated into the transplantation part of the trial

- Stage 1 is assessed following transplantation in 3 patients,
- Stage 2 is assessed after transplantation in 11 patients in total (combining stage 1 and stage 2).
- The final assessment point is reached after a total of 22 patients (combined stage 1, stage 2 and stage 3 transplants) have undergone transplantation. Each patient will be assessed for the primary end-point of survival at 90-days post transplantation.
-

DMC (or TSG – as appropriate) meetings will be held to discuss the results of each stage of the trial at appropriate time points.

The study endpoint is expected to be 6 months (180 days) after the last patient (patient 22) has received a liver transplant and have either reached the 6 month follow-up time point, have been withdrawn from the trial or through patient death, whichever is first. Long term follow-up visits are scheduled for 12 and 24 months post-transplant and data will continue to be collected at these time points.

The final analyses will be initiated once all data has been cleaned and queries resolved, following the study endpoint. The Trials Office will notify the main REC and MHRA that the trial has ended and a summary of the clinical trial report will be provided within 12 months of the end of trial. Supplementary reports may be provided to the MHRA at later dates regarding long-term patient survival.

1B.2: RECRUITMENT AND RANDOMISATION

1B.2.1 Recruitment

Please refer to section 1A.2.1

1B.2.2 Randomisation

Please refer to section 1A.2.2

1B.2.3 Ineligible Patients

Ineligible patients are defined as those registered patients who subsequently meet any of the exclusion criteria (Protocol section 4.5) and will be excluded from further participating in the VITTAL trial. Only patients who undergo transplant surgery will be included in the analysis.

Investigators may withdraw a registered patient from the trial in order to protect their safety and/or if they are unwilling or unable to comply with the required trial procedures (see Protocol section 6.11 for further details). Data will be collected from time of consent up to the time or withdrawal – this data will be used in the analysis providing consent is given by the patient.

1B.3: DATA QUALITY

Please refer to section 1A.3

1B.4: TRIAL POPULATIONS

1B.4.1 Baseline Characteristics

All collected baseline characteristics will be presented: for continuous measures the mean, standard deviation, minimum, maximum and IQR will be presented; for categorical variables the number and proportion per category will be presented.

The following patient demographics will be reported descriptively:

- Age
- Sex
- Aetiology of liver disease
- Indication of transplant
- Model for End-Stage Liver Disease (MELD) score (based on international normalised ratio (INR), creatinine, bilirubin)
- UKELD
- BMI
- A baseline assessment of quality of life using the EQ-5D-5L, performed time of consent to study (may coincide when patient was first put on liver transplant waiting list).

1B.4.2 Definition(s) of Populations for Analysis

All trial registered patients receiving an NMLP liver transplant will be included in the analysis population.

Patients who may have deviated from protocol will be reported as part of trial compliance. All recipients will be encouraged to complete the 12 and 24 month follow-up. Data from patients withdrawing or being withdrawn from the trial will be used in the analyses unless the patient requests otherwise – this will be stipulated on the trial consent form.

1B.5: TREATMENT RECEIVED

Patients are hospitalised immediately post-operation until day of discharge. Following transplantation, patients will be assessed daily by their clinical team and managed according to normal local protocols. Details of induction immunosuppression and maintenance immunosuppression (including doses) will be recorded according to protocol requirements.
Patient Resource Log to be given to patient at discharge or at next available clinic appointment.
Post-transplantation follow-up visits, where possible, will coincide with routine outpatient appointments. If the recipient is an inpatient, assessment will be made in hospital where appropriate.
The requirement for renal replacement therapy at any time post-transplantation will be recorded at follow-up visits.
All study participants will undergo magnetic resonance cholangiopancreatography (MRCP) with T2-weighted turbo-spin echo sequences at 6 months post-transplant unless contraindicated (as listed in the protocol).
Protocol deviations will be reported descriptively by type and number, no significance testing will be performed on these data.

1B.6: TOXICITY AND SAFETY ANALYSIS

All medical occurrences which meet the definition of an AE, Device Deficiencies and Adverse Device Effect (ADE), (see protocol Appendix 7 for definitions) will be reported. This also includes abnormal laboratory findings reported as clinically significant. All AEs will be reported in accordance with Clavien-Dindo classification of surgical complications (see protocol Appendix 1). A list of common general and disease/condition/surgery AEs is given in the protocol (protocol section 8.1).

All AEs, SAEs, Device Deficiencies, ADEs, abnormal laboratory findings will be reported by number of occurrence, proportion, grade, type and classification.

1B.7: ANALYSES OF OUTCOME MEASURES

1B.7.1 Definition and Calculation of Primary Outcome Measures

Achievement of successful transplantation of previously rejected donor livers following viability testing using NMLP, assessed using 90-day patient survival.

Calculated using:

$$90\ day\ patient\ survival\ rate = \frac{Number\ of\ patients\ alive\ at\ 90\ day\ post\ transplantation}{Total\ number\ of\ transplanted\ patients}$$

1B.7.2 Descriptive Analyses for the Primary Outcome Measures

The 90-day patient survival rate will be reported as a percentage, with the number of patients alive at 90-day post-transplantation in the numerator and the total number of trial transplantations performed in the denominators (e.g. calculated using the equation in section A7.1), together with a 95% confidence interval (Wilson (1927) method).

If possible, survival estimates calculated using Kaplan-Meier will be reported and 90-day survival with 95% confidence intervals and a plot will be produced.

1B.7.3 Hypothesis Testing for the Primary Outcome Measures

1B.7.3.1 Hypotheses Under Test

- Null hypothesis is that the 90-day patient survival rate will be less than or equal to 73%
- Alternative hypothesis is that the 90-day patient survival rate will be at least 88%

1B.7.3.2 Methods

- **Stage 1B of accrual:** 3 transplants using rescued livers will be assessed at the first stage. Using the hypothesis from section 1B.7.3.1, we would reject transplantation of rescued livers at the end of the first stage of accrual if only 1 or 0 patients survived †90-days post-transplantation. Otherwise, the trial proceeds into stage 2B.

- **Stage 2B of accrual:** An additional 8 transplants using rescued livers will be accrued (giving a combined total of 11 transplants from stage 1 plus stage 2). Using the hypothesis from section 7.3.1, we would reject transplantation of rescued livers at the end of the second stage of accrual if 7 or fewer patients had †90-day survival post-transplantation. Otherwise, the trial proceeds into stage 3B.

- **Stage 3B of accrual:** A further 11 transplantations using rescued livers will be accrued (giving an overall total of 22 transplants from stages 1, 2 and 3).The trial will conclude procedural success for transplants using rescued livers if there are at least 18 patients achieving †90-day survival.

†To reduce the suspension in recruitment whilst the interim analyses are being performed, the decision to stop or go on for the transplantation stage would be decided upon the graft survival and viability post-transplantation. This will be judged on the patient being released from hospital (usually between 10-14 days post-surgery). At each interim stage, all accumulated information on 90-day patient survival data that are available will be presented at the TSC meetings. Subsequent complete 90-day patient survival data for each interim stage will be reported to the TSC when they are available post the meeting. Additional TSC meetings will be conducted upon request if the success criteria is not met.

1B.7.3.3 Sample Size Calculations

The transplantation study using rescued marginal livers has been designed using an optimal three-stage design (Chen, 1997) testing a null hypothesis that patient 90-day survival is 73% against an alternative hypothesis that it is 88%, with 80% and 20% alpha. The total sample size requires 22 transplantations to be performed using NMLP rescued marginal livers.

1B.8: Secondary Outcome Measures Analyses

No hypothesis testing is planned for any secondary outcome measures, and any analyses will be mainly descriptive.

The secondary outcome measures will be compared with a contemporary matched recipient group.

The contemporary matched recipient group will be matched using the following:

- **Patient Characteristics** - Age, Sex, BMI, MELD, UKELD, Aetiology
- **Donor Livers Characteristics** – DCD or DBD, Sex

Secondary objectives and associated outcome measures are as follows:

1. **Objective:** Assess the liver graft function post-transplantation by incidence of primary non-function and early allograft dysfunction
   Outcome measures:
   - **Liver function tests** – data collected at every visit. Values will be reported as changes over time and plotted. If appropriate, the data may be modelled using an appropriate linear (or flexible model) mixed effects model (accounting for subject correlation). A comparison with the contemporary matched recipient group may be descriptive only. Liver function tests are: AST, ALT, GGT, Bilirubin and INR.
   - **30-day patient survival** – reported as a percentage with a 95% confidence interval (Wilson (1927) method). A comparison with the contemporary matched recipient group may be descriptive only.
   - **30-day graft survival** - reported as a percentage with a 95% confidence interval (Wilson (1927) method). A comparison with the contemporary matched recipient group may be descriptive only.
   - **90-day graft survival** - reported as a percentage with a 95% confidence interval (Wilson (1927) method). If possible survival estimates calculated using Kaplan-Meier will be reported for median and 90-day survival with 95% confidence interval and a plot will be produced. A comparison with the contemporary matched recipient group may be descriptive only.
   - **12-month patient survival** - survival estimates calculated using Kaplan-Meier will be reported for 12-month patient survival with 95% confidence interval and a plot will be produced. A comparison with the contemporary matched recipient group may be descriptive only.
   - **12-month graft survival** - survival estimates calculated using Kaplan-Meier will be reported for 12-month graft survival with 95% confidence interval and a plot will be produced. A comparison with the contemporary matched recipient group may be descriptive only.

2. **Objective:** Assess morbidity associated with receipt of NMLP rescued marginal livers
   - **Outcome measures:**
   - **Adverse event rates and severity:** these are graded according to the Clavien-Dindo classification (Protocol appendix 1). All data will be reported but specifically grade III complications and above will be reported in a separate table.
   - **Requirement of renal replacement therapy (renal injury)** – reported descriptively as a number and percentage.
   - **Ischemic cholangiopathy rate at 6 months** – reported descriptively as a number and percentage.
   - **Incidence of biliary complications** – reported descriptively as number and percentage of each particular type of biliary complication.

- **Incidence of vascular complications** – reported descriptively as number and percentage of each particular type of vascular complication.
- **Biopsy-proven acute rejection** – reported descriptively as number and percentage.
- **Reoperation rate** – reported descriptively as number and percentage
- **Length of intensive therapy unit stay** – reported descriptively as mean, standard deviation, minimum and maximum duration in days.
- **Length of hospital stay** – reported descriptively as mean, standard deviation, minimum and maximum duration in days.

3. **Objective:** Assess the physiological response of grafts to perfusion
Outcome measures:
    - **Post-reperfusion syndrome** - defined as a decrease in mean arterial pressure (MAP) >30% from the baseline for more than one minute during the first five minutes after reperfusion (assessed in the context of inotrope use). Here the number and percentage of liver grafts indicating post-perfusion syndrome may only be reported descriptively.

4. **Objective:** Identify the impact on quality of life after transplantation with NMLP rescued marginal livers
    - **Outcome measures:**
    - **Quality of life by delivery of the EQ-5D-5L questionnaire** (Protocol appendix 2) at baseline, day 30 and 6 months post-transplant – questionnaire data will be used to calculate any change (improvement or worsening) from baseline to day 30 and at 6 months. The calculation will be reported as the absolute value together with the percentage change to enable direct comparison for each patient. In addition, the data will be plotted as repeated measures over time to demonstrate graphically the behaviour over the treatment period, and will also be analysed using an appropriate repeated measures analysis method to assess if there is any trend over time.

1B.9: Additional Analyses and Exploratory Outcomes

1. **Serum Biochemistry Results:** the end point of trial participation is 6 months however serum biochemistry data will continue to be collected from consenting patients at 12 and 24 months post-transplantation (collected as part of routine care).

The biochemistry parameters recorded are:

- Serum bilirubin (μmol/l)
- Serum gamma-glutamyl transferase (GGT) (IU/L)
- Serum aspartate transaminase (AST) (IU/L)
- International normalised ratio (INR)

Descriptive statistics will be reported along with changes over time, and plots showing measurements over time for each patient. If appropriate, the data may be modelled using an appropriate linear (or flexible) mixed effects model (accounting for subject correlation).

2. **Work Package 2:** It is not planned for the trial statistician to be involved in any exploratory outcomes analyses relating to Work Package 2, other than in an advisory capacity. Work Package 2 is described in the protocol in section 2.2.3.

1B.10: Decision Criteria

Using an optimal three-stage trial design (Chen, 1997):

Stage 1B of accrual: Following transplantation in 3 patients, the trial will stop early (concluding P≤0.73) if there are 1 or 0 patients achieving 90-day survival, otherwise proceed to stage 2B.

Stage 2B of accrual: After transplantation in 11 patients (combined first and second stages) the trial will stop early (concluding P≤0.73) if there are seven or fewer successes, otherwise proceed to stage 3B.

Stage 3B of accrual: After transplantation in 22 patients in all three stages, the trial will conclude successful achievement of successful transplantation of previously rejected donor liver following NMLP if there are at least 18 patients achieving 90-day survival. If there are 17 or fewer patients achieving 90-day survival the method will be concluded as unsuccessful.

1B.11: Subgroup Analysis

- No subgroup analyses are planned.

SECTION 2: FURTHER ANALYSIS INFORMATION

2.1. HEALTH ECONOMICS
No health economic analyses will be performed.

2.2. STATISTICAL SOFTWARE
Stata version 14.1, StataCorp, Texas, USA.

2.3. STORAGE AND ARCHIVING
Snapshots of data used in interim and final analyses, programs and analyses will be stored and archived in relevantly labelled files in the following location: CRCTU Shared Area (S:)/D3B/Shared/ Liver-NIHR/ ACADEMIC/UoB Sponsor/2.Open/VITTAL/Statistics.

2.4. REFERENCES
Simon R. Optimal two-stage designs for phase II clinical trials. Control Clin Trials. 1989; 10(1):1-10. PubMed PMID: 2702835.

Chen TT. Optimal three-stage designs for phase II cancer clinical trials. Statistics in Med. 1997; 16(23):2701-11. PubMed PMID: 9421870.

Wilson EB. Probable Inference, the Law of Succession, and Statistical Inferences. Journal of the American Statistical Association. 1927; 22(158): 209-212.
