# Supplementary material for: Transplantation of discarded livers following viability testing with normothermic machine perfusion
Source: Nat Commun. 2020 Jun 16;11:2939. doi: 10.1038/s41467-020-16251-3 (PMC7298000; doi:10.1038/s41467-020-16251-3)
Supplement: Supplementary file 3 — Reporting Summary [file 41467_2020_16251_MOESM3_ESM.pdf]

naturereseach

Corresponding author(s): Mr Hynek Mergental, Prof Darius Mirza

Last updated by author(s): Oct 27, 2019

Reporting Summary

Nature Research wishes to improve the reproducibility of the work that we publish. This form provides structure for consistency and transparency in reporting. For further information on Nature Research policies, seeAuthors & Referees and theEditorial Policy Checklist .

Statistics

For all statistical analyses, confirm that the following items are present in the figure legend, table legend, main text, or Methods section.

n/a	Confirmed	
☐	☒	The exact sample size (n) for each experimental group/condition, given as a discrete number and unit of measurement
☐	☒	A statement on whether measurements were taken from distinct samples or whether the same sample was measured repeatedly
☐	☒	The statistical test(s) used AND whether they are one- or two-sided<br>*Only common tests should be described solely by name; describe more complex techniques in the Methods section.*
☐	☒	A description of all covariates tested
☐	☒	A description of any assumptions or corrections, such as tests of normality and adjustment for multiple comparisons
☐	☒	A full description of the statistical parameters including central tendency (e.g. means) or other basic estimates (e.g. regression coefficient) AND variation (e.g. standard deviation) or associated estimates of uncertainty (e.g. confidence intervals)
☐	☒	For null hypothesis testing, the test statistic (e.g. F, t, r) with confidence intervals, effect sizes, degrees of freedom and P value noted<br>*Give P values as exact values whenever suitable.*
☒	☐	For Bayesian analysis, information on the choice of priors and Markov chain Monte Carlo settings
☒	☐	For hierarchical and complex designs, identification of the appropriate level for tests and full reporting of outcomes
☐	☒	Estimates of effect sizes (e.g. Cohen's d, Pearson's r), indicating how they were calculated

Our web collection on statistics for biologists contains articles on many of the points above.

Software and code

Policy information about availability of computer code

Data collection	The data was collected in an electronic case report form coded for the trial.
Data analysis	STATA software package version 15.1 for Windows (StataCorp LLC, USA)

For manuscripts utilizing custom algorithms or software that are central to the research but not yet described in published literature, software must be made available to editors/reviewers. We strongly encourage code deposition in a community repository (e.g. GitHub). See the Nature Research guidelines for submitting code & software for further information.

Data

Policy information about availability of data

All manuscripts must include a data availability statement. This statement should provide the following information, where applicable:

- Accession codes, unique identifiers, or web links for publicly available datasets
- A list of figures that have associated raw data
- A description of any restrictions on data availability

The source data underlying figures and tables included in the main manuscript are provided within a Source Data file and supplementary tables. Additional data would be provided upon request.

The protocol design/methodology has been published in the BMJ Open : Laing RW, Mergental H,Yap C, et al. Viability testing and transplantation of marginal livers (VITTAL) using normothermic machine perfusion: study protocol for an open-label, non-randomised, prospective, single-arm trial. BMJ Open 2017;7:e017733. doi:10.1136/bmjopen-2017-017733

Field-specific reporting

Please select the one below that is the best fit for your research. If you are not sure, read the appropriate sections before making your selection.

- [x] Life sciences
- [ ] Behavioural & social sciences
- [ ] Ecological, evolutionary & environmental sciences

For a reference copy of the document with all sections, see nature.com/documents/nr-reporting-summary-flat.pdf

Life sciences study design

All studies must disclose on these points even when the disclosure is negative.

Sample size

The VITTAL Trial contains two parts: 1. Transplantation component required 22 patients to undergo transplantation based on an optimal three-stage design using alpha (type 1 error) and beta (type 2 error) of 0.2 and null of 0.73 versus 0.88 (Chen, 1997); and 2. Perfusion component required 53 livers to be perfused based on a two-stage design using target alpha of 0.05 (actual alpha = 0.047), target beta of 0.10 (actual beta = 0.098) and null of 0.30 versus 0.50 (Simon, 1989).

Data exclusions

No data were excluded from the analyses.

Replication

Primary outcome results were replicated by a second member of the biostatistics team at the Cancer Research Clinical Trials Unit, University of Birmingham.

Randomization

No randomization was applied in this trial.

Blinding

Blinding was not applicable to this trial.

Reporting for specific materials, systems and methods

We require information from authors about some types of materials, experimental systems and methods used in many studies. Here, indicate whether each material, system or method listed is relevant to your study. If you are not sure if a list item applies to your research, read the appropriate section before selecting a response.

Materials & experimental systems

n/a	Involved in the study	
x		Antibodies
x		Eukaryotic cell lines
x		Palaeontology
x		Animals and other organisms
	x	Human research participants
	x	Clinical data

Methods

n/a	Involved in the study	
x		ChIP-seq
x		Flow cytometry
x		MRI-based neuroimaging

Human research participants

Policy information about studies involving human research participants

Population characteristics

The patients undergoing transplantation were 63.6% male and 36.4% female; median age 56 years (IQR 46 to 65 years); median BMI 28.5 (IQR 24.0 to 31.0); median UKELD 52 (IQR 49 to 55); median MELD 12 (IQR 9 to 16); Transplantation indications (a patient may have more than one indication)- 36.4% alcohol-related liver disease, 18.2% non-alcohol steatohepatitis, 9.1% Hepatitis C virus, 9.1% primary biliary cirrhosis, 27.3% primary sclerosing cholangitis, 13.6% hepatocellular carcinoma, 4.5% need for intra-operative CVVH.

Recruitment

Patients were eligible if listed electively for primary liver transplantation and deemed to be low to moderate transplant risk candidates, suitable to receive a high-risk graft, as assessed by the Unit's transplant waiting list multi-disciplinary team. Candidates were required to have a patent portal vein, no significant cardiovascular co-morbidities, a UKELD score of 62 or less and no history of major upper abdominal surgery. Each patient was fully informed in advance of being offered a marginal graft and gave written consent for the trial. Patients were selected for transplantation when a suitable liver was available.

Ethics oversight

The trial was granted approval by the National Research Ethics Service in London-Dulwich (REC reference 16/LO/1056, Protocol number RG 15-240) and the Medicines and Healthcare Products Regulatory Agency.

Note that full information on the approval of the study protocol must also be provided in the manuscript.

Clinical data

Policy information about clinical studies

All manuscripts should comply with the ICMJE guidelines for publication of clinical research and a completed CONSORT checklist must be included with all submissions.

Clinical trial registration	ClinicalTrials.gov NCT02740608
Study protocol	The protocol has been previously published: Laing et al. BMJ Open 2017;7(11):e017733, and has been included in the appendix of this manuscript.
Data collection	All transplant recipient patients were registered at the Queen Elizabeth Hospital, Birmingham. Relevant trial data were recorded at each scheduled trial visit as stipulated in the trial protocol (days: 0, 30, 90, 180 and 12 and 24 months). Perfusion data were recorded at the Queen Elizabeth Hospital, Birmingham at time of perfusion. Liver donor data were recorded at the hospital where the donated liver registered and collected from.
Outcomes	The Trial has two linked primary outcomes: A) Establish the feasibility of NMP-L to increase the number of transplantable livers - assessed by the proportion of rejected livers than can be used for transplantation having been deemed viable following a period of machine perfusion; and B) Achieve successful transplantation of previously rejected donor livers following viability testing using NMP-L - assessed by 90-day patient survival, calculated as the number of patients alive 90-days post-NMP-L treated marginal liver transplantation (numerator) divided by the total number of NMP-L treated marginal liver transplants performed (denominator). Secondary outcome measures are: Assessment of liver graft function following transplantation by incidence of primary non-function and early allograft dysfunction; Liver function tests, 90-day graft survival; 12-month patient and graft survival; Adverse event rates and severity, graded by Clavien-Dindo classification; occurrence of post-reperfusion syndrome, assessed by he use of inotrope; impact on quality of life using EuroQoL5 questionnaires completed pre- and post-transplantation.
